# Treatment of antibiotic-manufacturing wastewater enriches for *Aeromonas veronii*, a zoonotic antibiotic-resistant emerging pathogen

**DOI:** 10.1093/ismejo/wraf077

**Published:** 2025-04-21

**Authors:** Xingshuo Wang, Meilun Wang, Wei Zhang, Hui Li, James M Tiedje, Jizhong Zhou, Edward Topp, Yi Luo, Zeyou Chen

**Affiliations:** College of Environmental Science and Engineering, Ministry of Education Key Laboratory of Pollution Processes and Environmental Criteria, Nankai University, Tianjin 300071, China; College of Environmental Science and Engineering, Ministry of Education Key Laboratory of Pollution Processes and Environmental Criteria, Nankai University, Tianjin 300071, China; Department of Plant, Soil and Microbial Sciences, Michigan State University, East Lansing, MI 48824, United States; Department of Plant, Soil and Microbial Sciences, Michigan State University, East Lansing, MI 48824, United States; Department of Plant, Soil and Microbial Sciences, Michigan State University, East Lansing, MI 48824, United States; Institute for Environmental Genomics and Department of Microbiology and Plant Biology, University of Oklahoma, Norman, OK 73019, United States; Agroecology Research Unit, Bourgogne Franche-Comté Research Centre, National Research Institute for Agriculture, Food and the Environment, Dijon 35000, France; State Key Laboratory of Water Pollution Control and Green Resource Recycling, School of the Environment, Nanjing University, Nanjing 210093, China; College of Environmental Science and Engineering, Ministry of Education Key Laboratory of Pollution Processes and Environmental Criteria, Nankai University, Tianjin 300071, China

**Keywords:** antibiotic manufacturing, manufacturing wastewater treatment, antibiotic resistance, pathogen, *Aeromonas veronii*

## Abstract

Antibiotic-manufacturing wastewater treatment plants primarily target chemical pollutants, but their processes may select for antibiotic-resistant pathogens and antibiotic resistance genes. Leveraging the combined strengths of deep metagenomic sequencing, 16S rRNA gene sequencing, quantitative polymerase chain reaction, and bacterial culturing, we investigated bacterial communities and antibiotic resistomes across eleven treatment units in a full-scale antibiotic-manufacturing wastewater treatment plant processing wastewater from a *β*-lactam manufacturing facility. Both bacterial communities and antibiotic resistance gene compositions varied across the treatment units, but were associated. Certain antibiotic resistance gene persisted through treatment, either carried by identical bacterial species, or linked to mobile genetic elements in different species. Despite the satisfactory performance in chemical removal, this plant continuously enriched zoonotic antibiotic-resistant *Aeromonas veronii* (an emerging pathogen responsible for substantial economic losses in aquaculture and human health) from influent to effluent, probably due to prolonged *β*-lactam selection pressure and aquatic nature of *A. veronii*. This enrichment resulted in a significantly higher abundance of *A. veronii* than other aquatic samples worldwide. Furthermore, the closest evolutionary relative to the retrieved *A. veronii* was an isolate obtained from the stool of a local diarrhea patient. These findings highlighted a substantial public health risk posed by antibiotic-manufacturing wastewater treatment, underlining its potential role in enriching and disseminating zoonotic antibiotic-resistant pathogens. Beyond chemical monitoring, enhanced surveillance of antibiotic-resistant pathogens and antibiotic resistance genes is needed in effluent discharge standard for antibiotic-manufacturing wastewater treatment plants.

## Introduction

The selection of antibiotic resistance in bacteria upon antibiotic exposure is a natural evolutionary phenomenon [1], which has been accelerated by the extensive use of antibiotics by humans [2], posing a significant threat to public health. Antibiotics are indispensable societal resources due to their powerful potency in treating bacterial infections [3], yet their manufacturing is resource-intensive, requiring substantial water use, and generating ⁓500–6500 m^3^ of wastewater per ton of antibiotics [4]. Global annual antibiotic production of 100–200 kilotons generates an estimated 50–1300 million cubic meters of wastewater [5]. Antibiotic manufacturing wastewaters are characterized by high organic content, significant biological toxicity, and elevated concentrations of antibiotic residues [6], often necessitating complex processing steps such as adsorption, biological treatment, membrane processes, and advanced oxidation to remove contaminants [7]. Despite advanced treatment, substantial quantities of antibiotic-resistant bacteria (ARB) and antibiotic resistance genes (ARGs) persist in antibiotic-manufacturing wastewater treatment plant (AM-WWTP) effluents [8, 9], posing significant health risks to both humans and animals. Therefore, AM-WWTPs offer a unique opportunity to study the dynamics of antibiotic resistance evolution across multistages and their health consequences.

Despite decades of concern about antibiotic resistance in AM-WWTPs and extensive research into this problem [8, 10, 11], previous studies have not comprehensively analyzed the dynamics of bacterial communities, ARB, and ARGs across different treatment stages in AM-WWTPs. This gap is largely due to the limitations in sample size, low-depth metagenomic sequencing (typically <12 Gb per sample), and traditional culturing methods, which often fail to detect low-abundance or nonculturable bacteria [12]. The present study harnessed the combined strengths of deep metagenomic sequencing, 16S rRNA gene amplicon sequencing, quantitative polymerase chain reaction (qPCR), and bacterial culturing to discover previously unknown low-abundance ARB and ARGs throughout a 11-unit treatment train of a full-scale AM-WWTP in anonymous Province, China. Additionally, comparative genomic analysis was used to assess the potential link between the AM-WWTP-borne resistant pathogen and clinical bacterial infections.

## Materials and methods

### Sample collection

**Figure 1:**
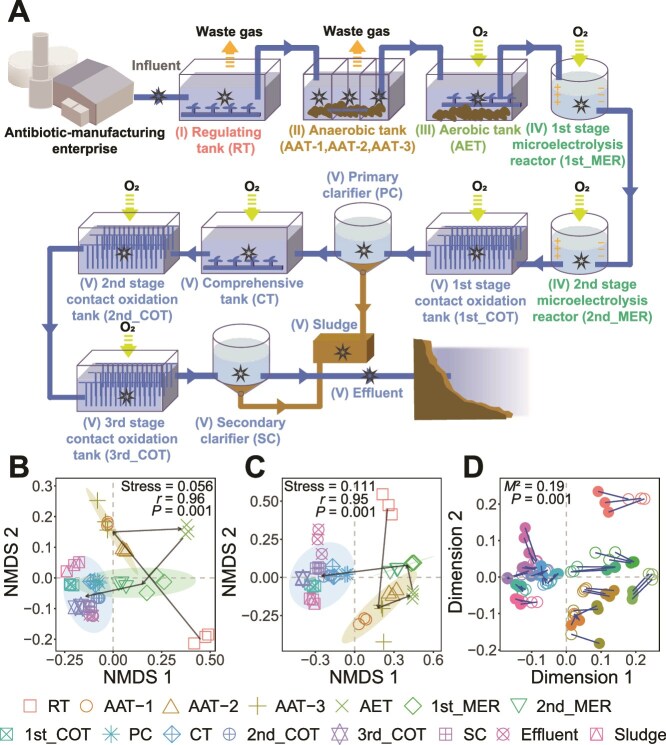
Sampling locations and analysis of ARGs and bacterial communities in a full-scale AM-WWTP. (A) Schematic description of sampling locations in a full-scale AM-WWTP. The sampling locations are marked with stars. Roman numbers I-V group the treatment units based on subsequent ARG and bacterial community analyses. NMDS analysis of (B) ARGs and (C) bacterial communities across all samples, clustered by different group. Groups I-V were formed based on a similar composition tested by anosim analysis, and arrows indicate the unidirectional flow from influent to effluent. Groupings of treatment units: Group I: Regulating tank (RT); group II: Anaerobic tank (AAT-1, AAT-2, AAT-3); group III: Aerobic stage (AET); group IV: Microelectrolysis reactors (1st_MER and 2nd_MER); group V: Oxidation tanks (1st_COT, 2nd_COT, and 3rd_COT), comprehensively regulating tank (CT), sediment tanks (PC and SC), effluent, and sludge. (D) Procrustes analysis showing the correlation between ARG profiles and bacterial community composition. Solid and hollow circles represent bacterial communities and ARGs, respectively, with circles from the same sample connected by blue lines.

The AM-WWTP from an antibiotic manufacturing enterprise situated in anonymous Province, China was selected for this research. This enterprise manufactures multiple oral and intravenous antibiotics through semi-synthetic modification, primarily *β*-lactams. A detailed list of the antibiotics produced is provided in the supporting Dataset S1. This AM-WWTP comprises 11 treatment units, combining conventional biological treatment with advanced microelectrolysis technology to process 600 m^3^ of wastewater daily (Fig. 1A). Sampling was conducted at 16 locations, marked by stars in Fig. 1A, with triplicate samples collected at each site. Wastewater samples were collected in 25-L polyethylene terephthalate (PET) buckets using a water sampler, while sludge samples were taken in 500-mL recoverable bags from the sludge storage tank. All samples were promptly transported to the laboratory on ice for DNA extraction, antibiotic quantification, and physicochemical analysis.

### Metagenomic sequencing

DNA extraction from the wastewater samples involved filtering appropriate volumes, as detailed in Dataset S2, through 0.22-μm membranes. Total DNA was then extracted from the filtered membranes using the DNeasy PowerSoil Pro Kit (Qiagen, DEU), following the manufacturer’s protocol. For sludge samples, DNA was extracted from 0.5 g of sludge. The extracted DNA was sent to Novogene Bioinformatics Technology Co., Ltd. (Beijing, China) for sequencing. DNA quality control, concentration determination, library construction, and data acquisition followed the previously described method [13]. Deep metagenomic sequencing was performed on a HiSeq 4000 System (Illumina), generating ⁓20 Gb of raw data per sample. All sequence data have been deposited in the NCBI Sequence Read Archive under accession number PRJNA1118895.

The detailed workflow for metagenomic sequence analysis is illustrated in Fig. S1 in the supporting information (SI). Briefly, raw reads were quality-filtered to generate high-quality paired clean reads using fastp (v0.20.1) with default filtering parameters [14]. Adapter sequences were automatically detected and trimmed. Reads were discarded if they met any of the following criteria: (i) ≥40% of bases with a phred quality score ≤15, (ii) ≥5 ambiguous nucleotides, or (iii) length <15 nt. The ARGs and mobile genetic elements (MGEs) in the clean reads were annotated and quantified using the ARGs-OAP pipeline, referencing the SARG v2.2 [15] and MGE database [16], respectively. Taxonomy annotation and quantification of reads were performed using MetaPhlan (v3.0.10) [17]. Megahit (v1.2.9) was used to assemble the reads into contigs using default parameters (the kmer size list included 21, 29, 39, 59, 79, 99 119 141, and only contigs ≥200 nt were retained) [18]. The open reading frames (ORFs) of all contigs were predicted using Prodigal (v2.6.3) and screened for ARGs via BLAST against the SARG v2.0 database (criteria: e-value ≤10^−5^, identity ≥70%, and query coverage ≥80%). ARG-carrying contigs (ACCs) were assigned to bacterial hosts at the species level using Kraken2 (v2.1.2) with the Minikraken v2 database [19]. To analyze mobile ARGs, all ACCs were annotated for MGEs using BLASTn (v2.13.0+) against the MGE database [20]. Contigs carrying both ARGs and MGEs were manually identified and recorded.

To acquire metagenome-assembled genomes (MAGs), metagenomic sequencing reads were processed using relevant modules of metaWRAP (v1.2.1) [21]. The completeness and contamination of each MAG were assessed with CheckM v1.1.2 [22], retaining only MAGs with ≥70% completeness and <10% contamination for further analysis. Mapping rates for both filtered contigs and MAGs were calculated by bwa [23], yielding 74.2%–89.1% for contigs and 30.1%–51.4% for MAGs (Dataset S3). The relative abundance of each MAG was calculated by normalizing the number of reads (based on average coverage) aligned to the MAG against the total number of reads in each sample [24]. Taxonomic assignment of MAGs was performed using GTDB-Tk (v1.5.0) based on the GTDB database release 202 [25]. ARGs carried by the MAGs were identified using a modified version of Plascad (v1.17) [26], while MGEs associated with MAGs were annotated via BLASTn (v2.13.0+) against the MGE database [20]. ORF prediction for MAGs was performed using Prodigal (v2.6.3) [27], and virulence factors (VF) were identified with diamond (v2.0.6.144) [28] against the VFDB (2019) database [29], applying the criteria: e-value ≤10^−5^, identity ≥70%, and query coverage ≥80%.

### 16S rRNA gene sequencing and absolute quantification of *Aeromonas veronii* using qPCR

To analyze bacterial communities across all samples, the V4 region of the 16S rRNA gene was amplified and sequenced, following our previous study [30]. Additionally, qPCR was performed to quantify the absolute abundance of *Aeromonas veronii* throughout the AM-WWTP process. Validated primers targeting *rpoB* housekeeping gene of *A. veronii* (Forward: CGTGCCGGCTTTGAAGTC; Reverse: GATCACGTACTTGC CTTCTTCAATA) were used. The qPCR procedure followed the previously described protocol [31].

### Isolation, identification, and characterization of *A. veronii*

Aeromonas Agar Base supplemented with 5 mg/L ampicillin (HepoBio, CHN) was used to isolate *A. veronii* at 37°C for 24 h. Single colonies were selected and re-cultured under the same conditions to obtain purified strains. Each strain was separately inoculated in Lysogeny Broth (LB) medium, incubated, and preserved at −80°C in LB with 20% (v/v) sterile glycerol. Genomic DNA was extracted using a Bacterial DNA Kit (Tiangen, CHN). The 16S rRNA gene was amplified using universal primer pairs of 27F: 5′-AGAGTTTGATCATGGCTCAG-3′ and 1492R: 5′-TACGGT TACCTTGTTACGACTT-3′, following the PCR procedure described previously [32]. The amplicons were sequenced at Novogene Bioinformatics Technology Co., Ltd. (Beijing, China) and analyzed through NCBI for sequence homology. To confirm taxonomy, the *rpoB* gene of the identified *A. veronii* isolates was also amplified and sequenced. For further characterization, *A. veronii* isolates were cultured on various agar media, and LB-cultured *A. veronii* was selected for Transmission Electron Microscopy (TEM) analysis. In brief, bacteria in the exponential phase were fixed in 2.5% glutaraldehyde in phosphate buffer for 4 h, stained with 2% (w/v) phosphotungstic acid, and imaged using a HITACHI HT7700 Exalens (HITACHI, Tokyo, Japan) TEM at 100 kV.

### Phylogenetic analysis of global *A. veronii* genomes

To compare the phylogenetic relationships between the identified *A. veronii* MAGs and other globally available *A. veronii* genomes, 267 publicly available *A. veronii* genomes were downloaded from the NCBI Assembly database as of 23 September 2022. These genomes originated from diverse sources, including human, animal, and environmental samples worldwide (Dataset S4). A rooted phylogenetic tree was constructed using the *A. veronii* genomes, including the MAGs identified in this study, based on species-level marker genes, following the previously published method [33] with *Pseudomonas fluvialis* used as outgroup. The maximal sub-clade was defined at the inner node where genomes exhibited a significantly shorter phylogenetic distance to the recovered MAGs compared to those outside this node. To assess whether strains from the same source or location were more likely to be related, we performed intercept-only multinomial logistic regression models with and without incorporating phylogeny as a covariance matrix. Model comparisons were conducted using the *loo_compare* function from the *brms* package [34]. In addition, a standard logistic regression model that excluded phylogenetic information was used to evaluate the extent to which strain clustering was associated with source or location, with significance assessed via the Wald test. Average nucleotide identity (ANI) values between the *A. veronii* genomes were calculated using fastANI (v1.32) to validate the phylogenetic tree [35].

### Antibiotic quantification and physicochemical analysis

Antibiotic extraction methods were adapted from a previous study [36]. Briefly, Na_2_EDTA (0.1 g) was added to 100 mL of solution diluted from 50 mL of sewage sample, and the pH was adjusted to 5.0 with hydrochloric acid. Subsequently, the antibiotics were extracted using solid phase extraction (SPE) with SAX cartridges (20 mg, 3 mL, Waters) and HLB cartridges (500 mg, 6 mL, Waters), preconditioned with 5 mL of methanol and 5 mL of ultrapure water. Target antibiotics were eluted with 10 mL of eluent (V_Methanol_: V_Acetone_ = 17: 3), evaporated to dryness under a nitrogen stream, reconstituted in 1 mL of methanol, and filtered through a 0.22-μm membrane before analysis. For the sludge samples, 1 g of lyophilized sludge was milled and mixed with 0.1 g of NaF, followed by extraction with 10 mL of solution (V_Methanol_: V_Citratebuffer_: V_EDTA_ = 3: 2: 1, both citrate buffer and EDTA at 0.1 M). The mixture was ultrasonicated for 15 min, centrifuged at 6140 × *g* for 10 min, and the supernatants were collected. The extraction was repeated, and combined supernatants were mixed with 5 mL of hexyl hydride and centrifuged at 6140 × *g* for 10 min. The middle layer was diluted to 500 mL with ultrapure water, adjusted to pH 5.0, and concentrated via SPE as described above. A Waters ultra-performance liquid chromatography-tandem mass spectrometer (UPLC-MS/MS; XEVO TQ-MS Mass Spectrometer, Waters Corporation, Milford, MA, USA) was used to analyze 53 target antibiotics following the previously published protocol [37].

For the wastewater samples, physicochemical properties, including pH, color, 5-day biochemical oxygen demand (BOD_5_), chemical oxygen demand (COD), ammonia nitrogen (NH_3_-N), total nitrogen (TN), total phosphorus (TP), total organic carbon (TOC), and suspended solids (SS) were measured according to the methods recommended by the Ministry of Ecology and Environment of China (https://www.mee.gov.cn/ywgz/fgbz/bz/). Similarly, for sludge samples, pH, TN, TP, and TOC were analyzed.

### Statistical analysis

All statistical analyses, including calculation of the Shannon index, Bray–Curtis-based distance, nonmetric multidimensional scaling (NMDS) analysis, and analysis of similarity (ANOSIM), were performed to test the significance of the differences using the *vegan* package in the R program [38]. The Kruskal-Wallis test, followed by Dunn’s test for multiple comparisons, was used to assess statistical significance. Procrustes analysis was conducted to examine the correlations between ARGs and the bacterial communities. Partial least squares-path modeling analysis was performed to determine the direct and indirect effects of selected variables on ARGs and bacterial communities using the *plspm* package [39]. Redundancy analysis (RDA) was applied to identify key physicochemical variables influencing community and ARG compositions.

## Results and discussion

### Dynamic ARG profiles and bacterial communities throughout the AM-WWTP

We first confirmed that the AM-WWTP significantly reduced COD and BOD_5_ from ~2000 and 900 mg/L in the influent to 130 and 50 mg/L in the effluent, respectively (Fig. S2 and Dataset S5), complying with local sewage discharge limits (COD <500 mg/L and BOD_5_ < 300 mg/L). Other parameters, including color, TOC, pH, TN, and NH_3_-N also showed substantial reductions from influent to effluent (Fig. S2 and Dataset S5).

Deep metagenomic sequencing (~20 Gb of raw data per sample) was successfully performed, except for the influent samples, which had low microbial DNA content due to harsh conditions such as extreme pH and high pollutant concentrations (Dataset S5). The ARG profiles and bacterial communities in the AM-WWTP clustered into five distinct groups (I-V), as visualized by NMDS analysis (Fig. 1B and C). These groups were primarily differentiated by the treatment technologies employed. Groups I-III corresponded to the samples from the regulating, anaerobic, and aerobic tanks, respectively. Group IV comprised samples from two microelectrolysis reactors, while Group V included samples from downstream units such as the contact oxidation, comprehensive regulation, sedimentation tanks, as well as effluent and sludge. Procrustes analysis further revealed a significant correlation between ARG profiles and bacterial community composition (Fig. 1D). Thus, despite substantial variation among treatment units, bacterial communities and ARG compositions remained closely linked.

**Figure 2:**
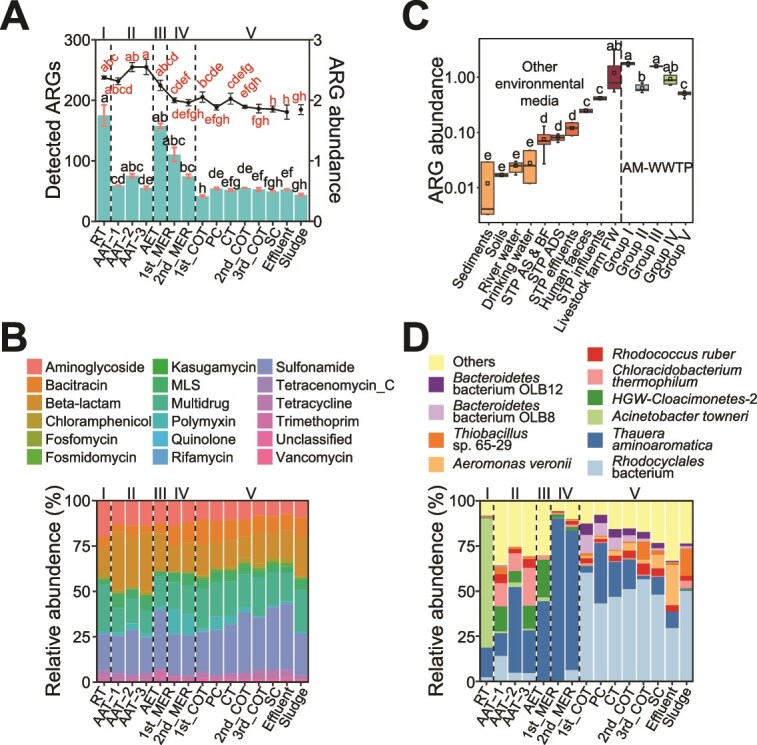
Dynamic ARG profiles and bacterial communities throughout the AM-WWTPs. (A) Total relative abundance of ARG subtypes (bar chart) and number of detected ARG subtypes (line chart). Roman numbers I-V group the treatment units based on ARG and bacterial community analyses. (B) Relative abundance of the dominant ARG types. (C) Comparison of ARG abundance in the studied AM-WWTP with 10 other environments, including STP, sewage treatment plants; AS&BF, activated sludge and biofilm; ADS, anaerobic digestion sludge; and livestock farm FW, feces and wastewater from livestock farms. Environments are grouped by total ARG abundance. (D) Abundance of bacteria at the species level.

A closer examination of the ARG profiles identified 444 ARG subtypes across 18 types, with *β*-lactam resistance being the most diverse, followed by multidrug, tetracycline, aminoglycoside, and macrolide-lincosamide-streptogramin (Additional file 1 in SI). The number of detected ARG subtypes generally decreased from the regulating tank to the effluent and sludge, although the anaerobic tank exhibited a higher number of ARGs than other units (Fig. 2A), likely due to its greater microbial biomass present in the anaerobic tank, as indicated by the subsequent bacterial community analysis. In terms of ARG abundance, the regulating tank had the highest levels, followed by a significant decrease in the anaerobic tank. ARG abundance then increased rapidly in the aerobic tank before consistently declining throughout the remaining treatment units. Overall, the AM-WWTP demonstrated moderate effectiveness in reducing ARG numbers and abundance. Sulfonamide (19.3%–36.3%) and *β*-lactam (11.8%–32.3%) resistance genes were the most abundant ARG classes detected (Fig. 2B), which corresponded to the dominant antibiotic types, sulfonamide and *β*-lactam, detected in the collected samples (Dataset S6). This suggests that the selective pressure exerted by antibiotic residues can promote the enrichment of corresponding ARG type. Moreover, compared with the Predicted No-Effect Concentrations (PNECs) for antibiotics established by the AMR Industry Alliance, which indicate thresholds above which resistance selection may occur [40], the dominant antibiotic concentrations detected exceeded their respective PENCs, further supporting their role in in situ selection of resistance genes. The microelectrolysis tanks exhibited significantly lower ARG α-diversity than other units, highlighting the strong performance of microelectrolysis treatment technique in ARG removal (Fig. S3A). Finally, a comparison of ARG abundance in the studied AM-WWTP with other natural and artificial environments, based on a previous study [41], showed that ARG levels were comparable to those in feces and wastewater from livestock farms, recognized hotspots for ARG, and significantly higher than in natural environments (Fig. 2C). This underscores the AM-WWTP's role as a major contributor to ARG pollution.

Regarding the microbial community, bacteria dominated all the tanks, with Methanobacteriaceae, a class of methanogenic archaea typically found in anoxic environments [42, 43], detected exclusively in the anaerobic tank (Fig. S3B). The most prevalent bacterial phylum throughout the treatment process was Proteobacteria, accounting for 55%–98.5% of the total abundance (Fig. S3C). α-diversity of the bacterial community followed this order: anaerobic tank (Group II) > Group V > aerobic tank (Group III) > regulating tank (Group I) > microelectrolysis tank (Group IV) (Fig. S3D). At the species level, certain bacteria were unique to specific tanks (Fig. 2D). For instance, *Acinetobacter towneri* dominated the regulating tank (71.6% in abundance), while *Thauera aminoaromatica* was prevalent in the microelectrolysis tank (83.7%). Additionally, *Rhodocyclales* bacteria dominaned Group V samples (29.1%–60.3%). This distinct species distribution likely contributed to the variation in ARG and bacterial community compositions across the treatment groups.

### Essential role of horizontal gene transfer of ARGs within AM-WWTP

**Figure 3:**
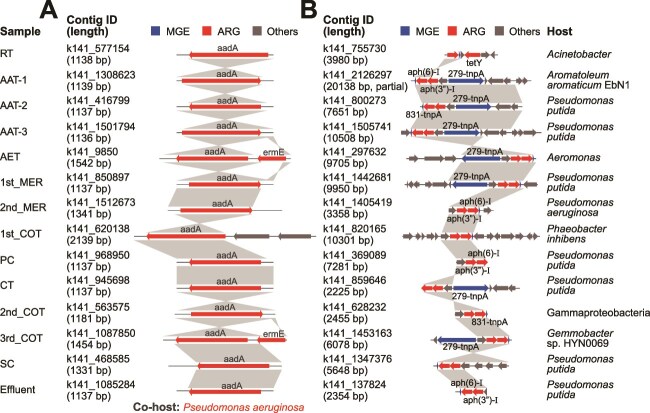
Fate of ARGs during the passage of the AM-WWTP. Certain ARG persisted through treatment, either carried by identical bacterial species, or linked to MGEs in different species. (A) Example of an ARG consistently hosted by the same bacterium across the AM-WWTP: *aadA* carried by *Pseudomonas aeruginosa*. Additional examples are provided in the Supplementary Additional File 2. (B) Physical linkage of certain ARGs with MGEs during the treatment process. One example shows different bacterial hosts carrying the ARG *tnpA* linked with the MGE *aph(6)-I*. Additional examples are shown in Fig. S6.

To assess the impact of environmental variables on ARGs and bacterial communities, redundancy analysis (RDA) was performed. It revealed that pH, NH_4_^+^-N, and COD were the top three influencing factors, collectively explaining ~60% of the variation in both ARGs and bacterial communities (Fig. S4). Nevertheless, 24.3% of ARG variation and 30.1% of bacterial community variation remained unexplained by the physicochemical properties, suggesting additional contributing factors. Given the significant correlation between ARGs and bacterial composition, sequence reads were assembled into contigs and identified ARG hosts, revealing 2502 ARG-carrying contigs (ACCs) across all samples. These ACCs contained 108 ARG subtypes spanning 13 classes, with bacitracin resistance gene *bacA*, the aminoglycoside resistance gene *aadA*, and the multidrug antibiotic resistance gene *ompR* being the most abundant (Dataset S7). Proteobacteria dominated the ARG hosts, harboring 2195 ARGs, followed by Bacteroidetes (104 ARGs) and Firmicutes (65 ARGs) (Fig. S5). Some AACs carrying identical ARGs in the same bacterial host were detected across all treatment units (Fig. 3A, Additional file 2), indicating their persistence throughout the process. Moreover, certain ARGs were physically linked to MGEs and were cohosted by different bacterial species across units. For instance, the aminoglycoside resistance gene *aph(6)-I*, the trimethoprim resistance gene *dfrA16*, and the tetracycline resistance gene *tetG* were associated with transposon *tnpA*, integrons *intI-1*, and *qacEdelta*, respectively (Figs. 3B and S6), highlighting the potential for HGT of ARGs within AM-WWTPs. Additionally, the proportion of ARG&MGE-co-carrying contigs in the AM-WWTP exceeded that in the pig, chicken, and human feces (Datasets S8 and S9), environments known for high microbial density and frequent HGT [44]. Partial least squares path modeling further underscored the crucial role of HGT, demonstrating that while antibiotic residues and bacteria influenced ARG profiles indirectly via MGEs, only MGEs had a significant direct impact on ARG persistence (Fig. S7). These findings collectively indicate that HGT plays an essential role in ARG persistence within AM-WWTPs, likely driven by high microbial densities, strong chemical stress, and biofilm-rich environments in the wastewater treatment process [45].

### Enrichment of the antibiotic-resistant pathogen *A. veronii*

**Figure 4:**
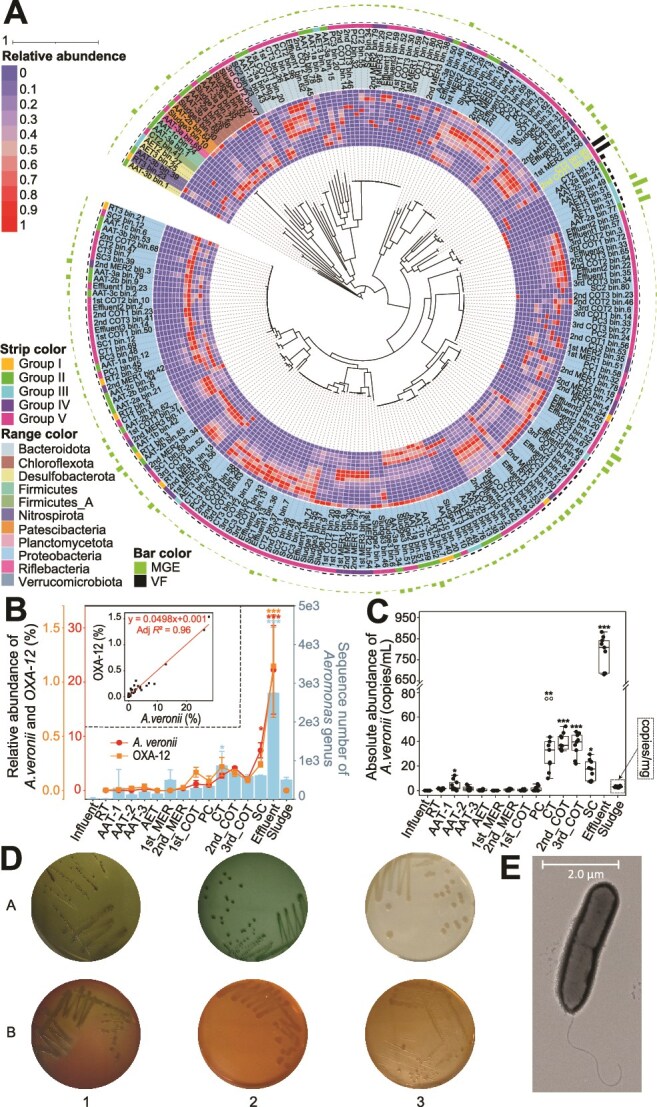
AM-WWTP enriches for *Aeromonas veronii*. (A) A phylogenetic tree at the center was built for ARG-harboring MAGs using a concatenated alignment of 120 universally distributed bacterial single-copy genes. Two *A. veronii* MAGs are highlighted. The standardized relative abundance of each MAG in all treatment units is shown in the heatmap in the 1st and 2nd annuli. Different 11 phyla are shown by range. The strip in the 3rd annulus denotes the corresponding treatment stage. The bar plot rings in the 4th and 5th annuli indicate the VF and MGE counts, respectively. (B) Line graph illustrating the relative abundance of reads-annotated *OXA-12* and *A. veronii* from the regulating tank to the effluent, showing a significantly correlation. Bar chart showing the consistent increase in *Aeromonas* genus abundance, based on 16S rRNA gene sequencing, from the influent to the effluent. Error bars represent standard deviation. Significant differences compared to the regulating tank (RT) are indicated as follows: ^*^  *P* < 0.05, ^**^  *P* < 0.01, ^***^  *P* < 0.001). (C) Absolute abundance of *A. veronii* quantified by qPCR during passage through the AM-WWTP. Significant differences from RT are showed. (D) Colony morphology of isolated *A. veronii* on various agar media plates (A1: Aeromonas Agar Base with 5 mg/L ampicillin; A2: Thiosulfate citrate bile salts sucrose agar; A3: Lysogeny broth agar; B1: Sheep blood agar; B2: MacConkey agar; B3: Salmonella-Shigella agar). (E) TEM micrograph of a single bacterium.

Metagenomic binning yielded 3336 high-resolution MAGs across all sequenced samples (Dataset S10), with 226 MAGs carrying ARGs, predominantly from Proteobacteria (137/226) (Fig. 4A). A total of 176 of these ARG-carrying MAGs also harbored virulence factors (VFs), suggesting the potential for both antibiotic resistance and pathogenicity. Among them, two *A. veronii* MAGs (2nd_COT1 bin.80 and CT2 bin.95) carried the highest number of VFs (59 and 44, respectively, Datasets S11 and S12) and exhibited a consistent increase in relative abundance from the regulating tank to the effluent (Fig. 4B). Metagenomic read-based annotation further revealed that both the relative abundance of *A. veronii* species and the *OXA-12* gene (one of two ARGs harbored by *A. veronii* MAGs) increased and correlated throughout the treatment process, from the regulating tank to the effluent (Fig. 4B). Additionally, 16S rRNA gene amplicon sequencing of all 48 samples, including the influent, confirmed this trend (Fig. 4B). qPCR quantification of the *rpoB* gene further corroborated this finding, showing a substantial increase in *A. veronii* during treatment (Fig. 4C), ruling out the possibility that the rise was an artifact of relative abundance in metagenomics or 16S rRNA gene sequencing. The largest enrichment occurred at the penultimate treatment step (from the secondary clarifier to the effluent) (Fig. 4B and C), likely due to the aquatic nature of *A. veronii* [46], which facilitates its release from suspended solids into liquid phases during sedimentation in the secondary clarifier. Supporting this hypothesis, *A. veronii* showed extremely low abundance in the collected sludge samples (Fig. 4B and C).

Two ARGs identified in the *A. veronii* MAGs were the bacitracin resistance gene *bacA* and *β*-lactamase gene *OXA-12* (Fig. S8). While the presence of *OXA-12* was anticipated, as most *Aeromonas* species produce *β*-lactamases [47], the discovery of *bacA*, encoding a protein involved in bacitracin resistance, was unexpected, given that bacitracin exclusively targets Gram-positive bacteria by disrupting their cell walls. In principle, Gram-negative *A. veronii* is intrinsically resistant to bacitracin, as the antibiotic’s large and complex structure prevents it from penetrating the outer membrane [48]. Alignment with clinical reference *A. veronii* FDAARGOS 632 genome revealed that *bacA* gene in the identified *A. veronii* genomes was located between the *gal* operon (involved in galactose metabolism) [49] and the *rpoE*–*chrR* operon (which regulates oxidative stress responses) [50] (Fig. S8). In this context, we speculate that *bacA* may serve an as yet cryptic function in *A. veronii*. The identified VFs in *A. veronii* MAGs were primarily associated with adhesion, secretion systems, and toxin production (Datasets S11 and S12).

Consistent with molecular findings, an antibiotic-resistant and potentially pathogenic *A. veronii* strain was isolated from the effluent (Fig. 4D and E). This strain exhibited a minimum inhibitory concentration of 325 mg/L to ampicillin (one representative *β*-lactam) and shared >99% homology in both the 16S rRNA and the *ropB* genes with a pathogenic *A. veronii* reference strain (Fig. S9). It displayed distinct colony morphologies: green edges with black centers on RYAN and TCBS media, white colonies on LB agar, and transparent pink colonies on MacConkey and SS agar (Fig. 4D). A clear hemolysis ring on blood agar (B1 in Fig. 4D) confirmed its pathogenic potential [51], while TEM imaging revealed a rod-shaped bacterium with a single polar flagellum (Fig. 4E).

Collectively, the findings demonstrate that the AM-WWTP facilitated the enrichment of antibiotic-resistant pathogen *A. veronii* from the influent to the effluent, despite its effective chemical pollutant removal. *A. veronii* is a zoonotic pathogen intrinsically resistant to *β*-lactams [52], posing risks to humans, fish, and other aquatic animals [46]. In humans, it can cause bacteremia, diarrhea, gastroenteritis, peritonitis, septicemia, wound infections, and urinary tract infections [46]. In fish, it is associated with abdominal distention, fin rot, exophthalmia, hemorrhagic septicemia, and ulceration, frequently leading to disease outbreaks and significant economic losses in aquaculture [53].

### High evolutionary similarity between AM-WWTP and clinically isolated *A. veronii*

**Figure 5:**
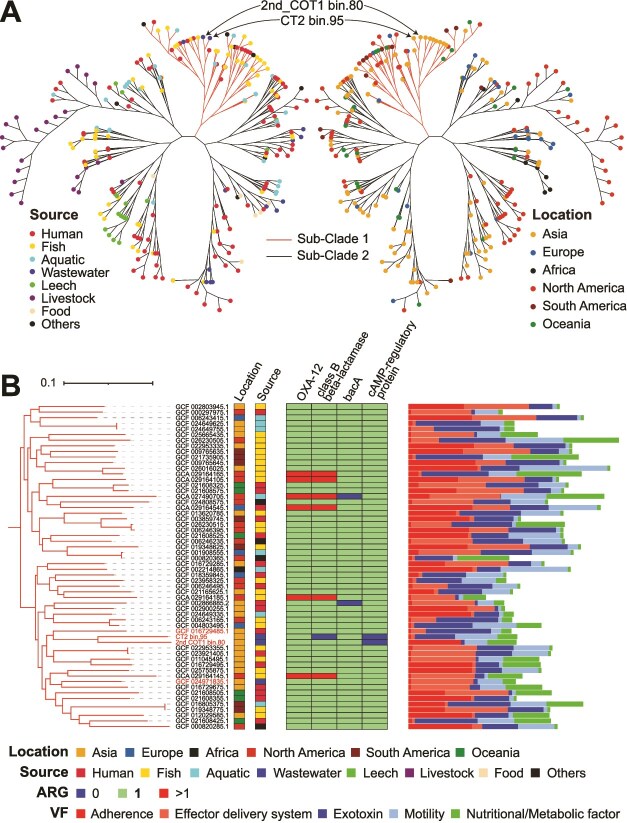
Phylogenetic analysis of global *Aeromonas veronii* genomes. (A) Phylogenetic tree of 267 downloaded and 2 recovered *A. veronii* genomes, categorized by the environmental source of isolation and the geographical locations of the samples. Sub-clade containing *A. veronii* recovered in this study is highlighted. (B) Detailed phylogenetic tree of this sub-clade, with two recovered *A. veronii* from AM-WWTP and two focal genomes from clinic marked. The heatmap displays ARG counts, and the stacked bar chart illustrates the VF gene number of each type.

To investigate the phylogenetic relationships among global *A.veronii* genomes, we constructed phylogenetic trees using 267 publicly available *A. veronii* genomes (Dataset S4) along with the two recovered in this study, categorized by sampling locations or environmental sources (Fig. 5A). Leave-one-out cross-validation comparison results indicate that strains from the same source or location are more likely to be related to each other, independently of neutral evolutionary processes (i.e. genetic variation arising primarily through random drift rather than selective pressures, Fig. S10 and Dataset S13). This finding suggests that *A. veronii* strains from the same source or location are more likely to share similar evolutionary characteristics. We further focused on sub-clade 1, which included the two recovered MAGs and primarily consisted of isolates from fish (25 genomes), humans (18 genomes), surface water (8 genomes), and wastewater (1 genome). Nearly all *A. veronii* genomes within this clade carried both multiple ARGs including *OXA-12*, *bacA*, class B *β*-lactamase, and cAMP-regulatory protein genes and VF genes with diverse mechanisms (Fig. 5B), highlighting the potential health risks associated with this subclade. The closest evolutionary relative to the two *A. veronii* from AM-WWTP was an isolate obtained from the stool of a patient with diarrhea (GCF 016729485.1, Fig. 5B) in the same province as the AM-WWTP [54, 55]. Genomic alignment revealed 96.3% similarity between the AM-WWTP-derived *A. veronii* and this clinical isolate (Fig. S11). Additionally, ARG alignment confirmed high degree of similarity in *OXA-12* and *bacA* genes between these strains (Fig. S12). Another wastewater-borne isolate (GCF 024971835.1) within sub-clade 1 was found in sewage from a Chinese hospital’s gastroenterology department [56] (Fig. 5B). Collectively, phylogenetic clustering and the presence of clinically relevant isolates in close geographical proximity highlight the potential health risk posed by the enriched *A. veronii* in the AM-WWTP effluent, warranting further epidemiological studies to confirm transmission pathways.

**Figure 6:**
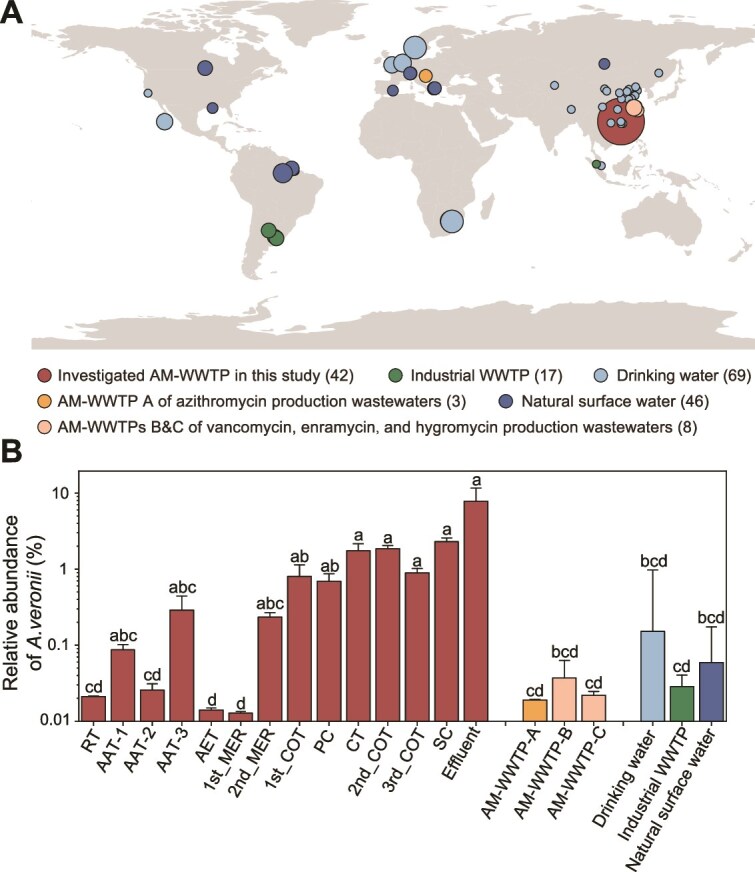
*Aeromonas veronii* enrichment was unique to the studied AM-WWTP. (A) Geographic distribution of samples collected from various aquatic habitats, with each point representing a sampling location. Point size corresponds to the number of samples collected, while point color indicates the habitat type. (B) Comparison of *A. veronii* abundance in the AM-WWTP with other aquatic environments on logarithmic scales. Bars are color-coded by habitat type, with error bars representing standard deviation. Statistical significance is indicated by letters, where different letters denote significant differences. Note: Abundance values in this figure were calculated using Kraken2, whereas other sections of the manuscript use Metaphlan. As a result, these values are not directly comparable to those presented elsewhere.

We compared *A. veronii* distribution in the studied AM-WWTP with various global water environments, including 69 drinking water, 17 non-antibiotic industrial wastewater, 46 natural surface water, and 11 wastewaters from AM-WWTPs receiving non-*β*-lactam antibiotics like azithromycin, vancomycin, enramycin, or hygromycin (Dataset S14 and Fig. 6A). *A. veronii* was significantly more abundant in the studied AM-WWTP receiving *β*-lactam wastewater than in all other water bodies (Fig. 6B), demonstrating its specific enrichment by *β*-lactams. The intrinsic resistance of *A. veronii* to *β*-lactams may enable the residual *β*-lactams to selectively promote its enrichment. Antibiotic residue analysis confirmed persistent low-levels of *β*-lactams across all treatment units (Fig. S13 and Dataset S6). Compared with the PNECs established by the AMR Industry Alliance to indicate the potential for resistance selection [40], these *β*-lactam residues exceed the corresponding PNEC thresholds (particularly cefixime, which reached levels tens to hundreds of times higher, Dataset S6) and are therefore sufficient to exert strong selective pressure on the microbial community, favoring the enrichment of *A. veronii*. The continuous increase of *A. veronii* throughout the treatment process likely reflects prolonged exposure and selection pressure exerted by *β*-lactams.

Overall, this study systematically characterizes bacterial communities and antibiotic resistomes across the entire treatment train of a full-scale AM-WWTP. It demonstrates that antibiotic selection pressure in an AM-WWTP receiving *β*-lactam-manufacturing wastewater can contribute to the enrichment and potential transmission of *A. veronii*, a zoonotic antibiotic-resistant pathogen responsible for significant economic losses in aquaculture and an increasing number of human infections [57]. The public health consequences associated with this enrichment in regions where such factories are located may warrant attention. Despite the satisfactory performance of the AM-WWTP in removing chemical pollutants, the effluent discharge may elevate transmission risks of *A. veronii* to both aquatic animals and humans. These findings also highlight the inadequacy of traditional monitoring and risk assessments that focus solely on chemical quality metrics in the AM-WWTP effluent. It is crucial to invest in and incorporate ARG and ARB surveillance into the wastewater management frameworks to better protect human and animal health from antibiotic-resistant pathogens.

## Supplementary Material

Supporting_information-final-20250416_wraf077(1)

Supporting_Datasets_wraf077(1)

## Data Availability

All sequence data generated in this study have been deposited in the NCBI sequence archive. The datasets used and/or analyzed in this study are available from the corresponding author upon reasonable request.
