# Supplementary material for: Treatment of antibiotic-manufacturing wastewater enriches for Aeromonas veronii, a zoonotic antibiotic-resistant emerging pathogen
Source: ISME J. 2025 Apr 21;19(1):wraf077. doi: 10.1093/ismejo/wraf077 (PMC12085271; doi:10.1093/ismejo/wraf077)
Supplement: Supporting_information-final-20250416_wraf077(1) [file supporting_information-final-20250416_wraf077(1).pdf]

Supporting information for

Treatment of antibiotic-manufacturing wastewater enriches for *Aeromonas veronii*, a zoonotic antibiotic-resistant emerging pathogen

Xingshuo Wang[1], Meilun Wang[1], Wei Zhang[2], Hui Li[2], James M Tiedje[2], Jizhong Zhou[3], Edward Topp[4], Yi Luo[5], and Zeyou Chen[1,*]

[1] College of Environmental Science and Engineering, Ministry of Education Key Laboratory of Pollution Processes and Environmental Criteria, Nankai University, Tianjin, 300071, China

[2] Department of Plant, Soil and Microbial Sciences, Michigan State University, East Lansing, Michigan, 48824, USA

[3] Institute for Environmental Genomics and Department of Microbiology and Plant Biology, University of Oklahoma, Norman, OK 73019, USA

[4] Agroecology Research unit, Bourgogne Franche-Comté Research Centre, National Research Institute for Agriculture, Food and the Environment, Dijon 35000, France

[5] State Key Laboratory of Water Pollution Control and Green Resource Recycling, School of the Environment, Nanjing University, Nanjing 210093, China

* Corresponding author: College of Environmental Science and Engineering, Ministry of Education Key Laboratory of Pollution Processes and Environmental Criteria, Nankai University, Tianjin, 300071, China. Email: zeyou@nankai.edu.cn

Figure S1. The bioinformatics analysis workflow.

Figure S2. Changes of selected physicochemical properties of the wastewater samples through all treatment units. (a) Suspended solids; (b) Colority; (c) pH; (d) Total organic carbon; (e) Chemical oxygen demand; (f) Biochemical oxygen demand for five days; (g) Total nitrogen; (h) Ammonia nitrogen; and (i) Total Phosphorus.

Figure S3. Microbial profiles of all the samples. (a) α-diversity indices of ARGs. (b) Abundance of microbes at the kingdom level; (c) abundance of bacteria at the phylum level; (d) α-diversity indices of bacterial communities.

Figure S4. RDA analysis of the relationships between environment variables with (a) bacteria and (c) ARGs, respectively. Red arrows represent the phylum level of bacteria and blue arrows represent environment factors. Total explained variance ratio were calculated and visualized in (b) and (d), respectively.

Figure S5. Hosts of ARGs at the phylum levels obtained from contigs statistical analysis.

Figure S6. Co-shared (a) *dfrA16*-ARG-carrying and (b) *tetG*-ARG-carrying contigs and their putative hosts in different samples.

Figure S7. (a) The Partial least squares-path modeling analysis showing the effects of antibiotics, physicochemical variables, MGEs, and bacteria on ARG compositions. The solid and dashed lines indicate the positive and negative effects, respectively. The numbers adjacent to each arrow are partial correlation coefficients at the significance level of *** ≤ 0.001, ** ≤ 0.01, or * ≤ 0.05. The R^2 values represent the proportion of variance explained for each factor. (b) The standardized total effect of antibiotics, physicochemical variables, MGEs, and bacteria on the ARG compositions.

Figure S8. Comparisons of (a) *bacA* and (b) *OXA-12* genes in two assembled *A. veronii* MAGs and *A. veronii* reference genome.

Figure S9. (a) NCBI Multiple Sequence Alignment of the 16S rRNA gene of the *A. veronii* obtained from the effluent and the most similar sequences. (b) Unrooted phylogenetic tree constructed by partial *ropB* gene of effluent obtained and some other isolated *A. veronii* strains.

Figure S10. (a) Branch length ignored phylogenetic tree to show inner nodes clearly. Nodes used in figure b is shown with circle. (b) Welch Two Sample t-test result of phylogenetic distance for genomes in sub-clade 1 and sub-clade 2 (right) and result of the parent node at the next level (left).

Figure S11. The circos map of A. veronii genomes' chromosome. Rings from inside to outside are: genome alignment results of (1) 2nd COT1 bin.80 vs reference genome (FDAARGOS 632), (2) CT2 bin.95 vs reference genome and (3) genome from diarrhea patient (GCF 016729485.1) vs reference genome; GC content of reference genome; GC skew of reference genome; genome annotation (+); genome annotation (-); positions related to ARG or HGT.

Figure S12. Multiple sequence alignment result for protein of *OXA-12* and *bacA*.

Figure S13. Concentration of the top 4 antibiotics in all wastewater samples. (a) Sulfonamides, (b) Quinolones, (c) *β*-lactams, and (d) Tetracyclines. Significance of differences is showed by letter. Error bars represent for Standard Deviation.

Additional file 1. Detailed ARG profiles in all samples.

Additional file 2. Some ARGs with identical host.

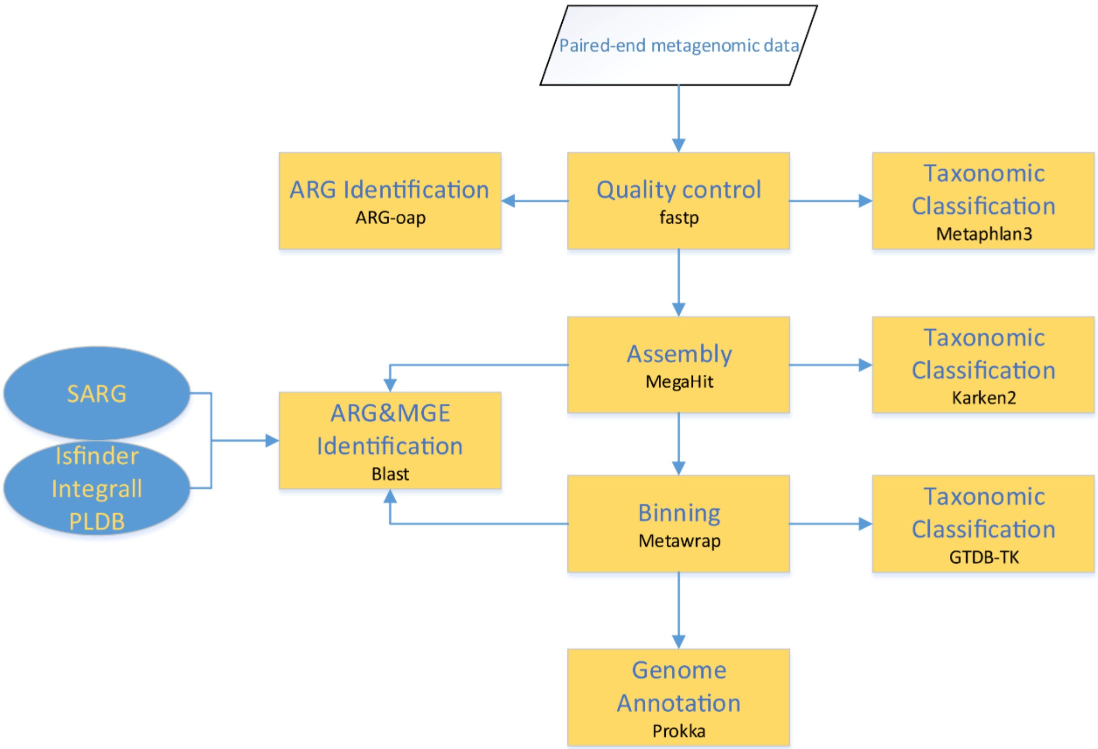

Figure S1. The bioinformatics analysis workflow.

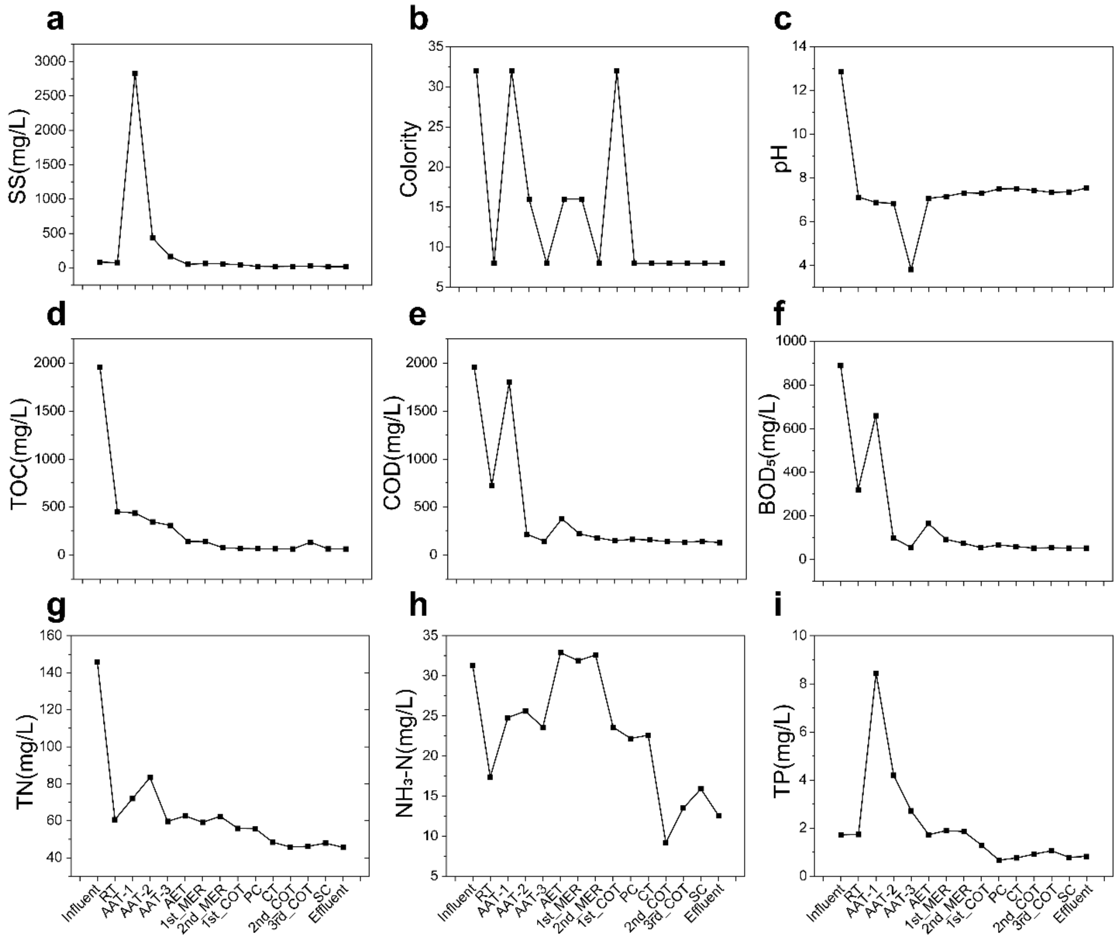

Figure S2. Changes of selected physicochemical properties of the wastewater samples through all treatment units. (a) Suspended solids; (b) Colority; (c) pH; (d) Total organic carbon; (e) Chemical oxygen demand; (f) Biochemical oxygen demand for five days; (g) Total nitrogen; (h) Ammonia nitrogen; and (i) Total Phosphorus.

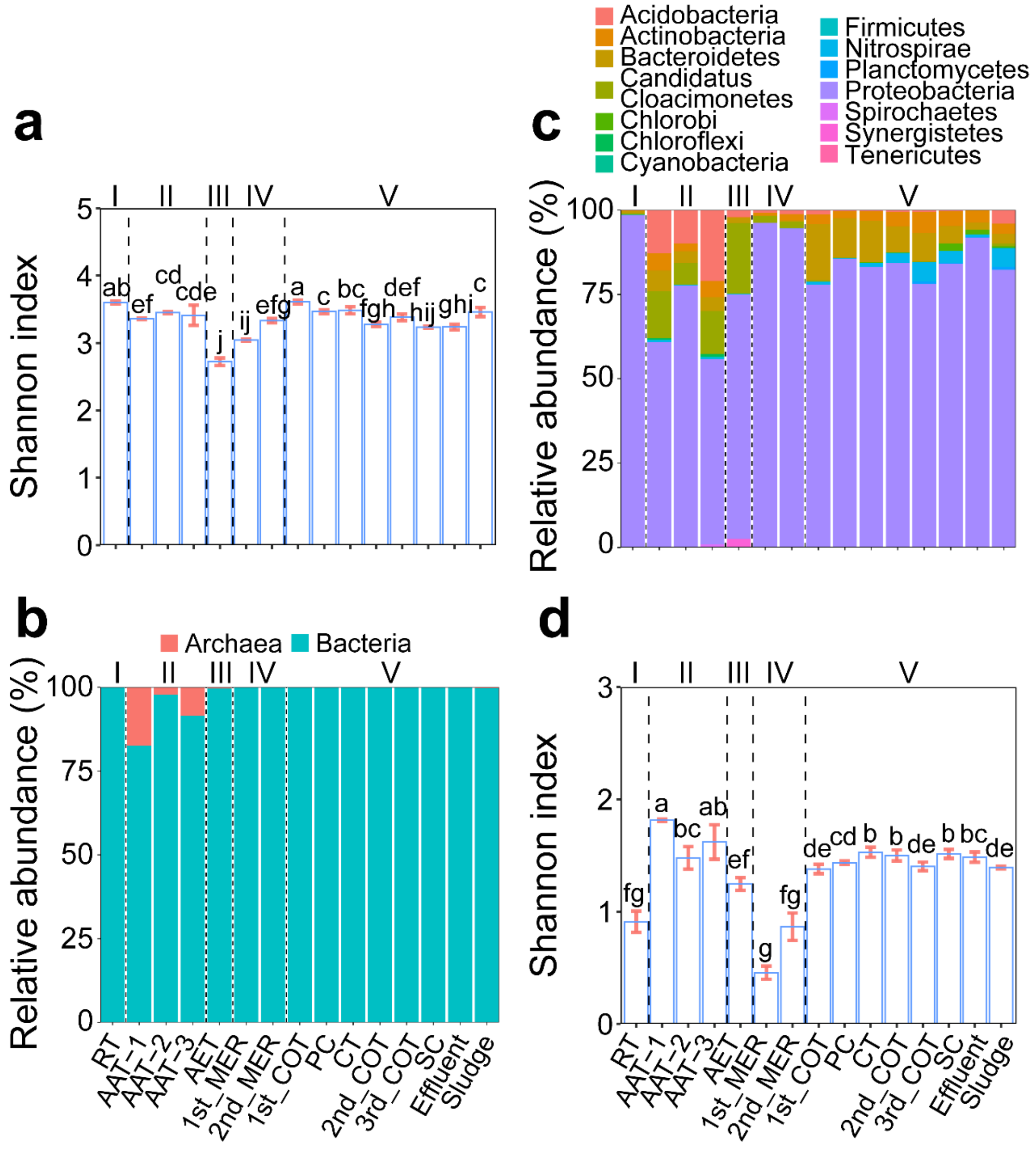

Figure S3. Microbial profiles of all the samples. (a) α-diversity indices of ARGs. (b) Abundance of microbes at the kingdom level; (c) abundance of bacteria at the phylum level; (d) α-diversity indices of bacterial communities.

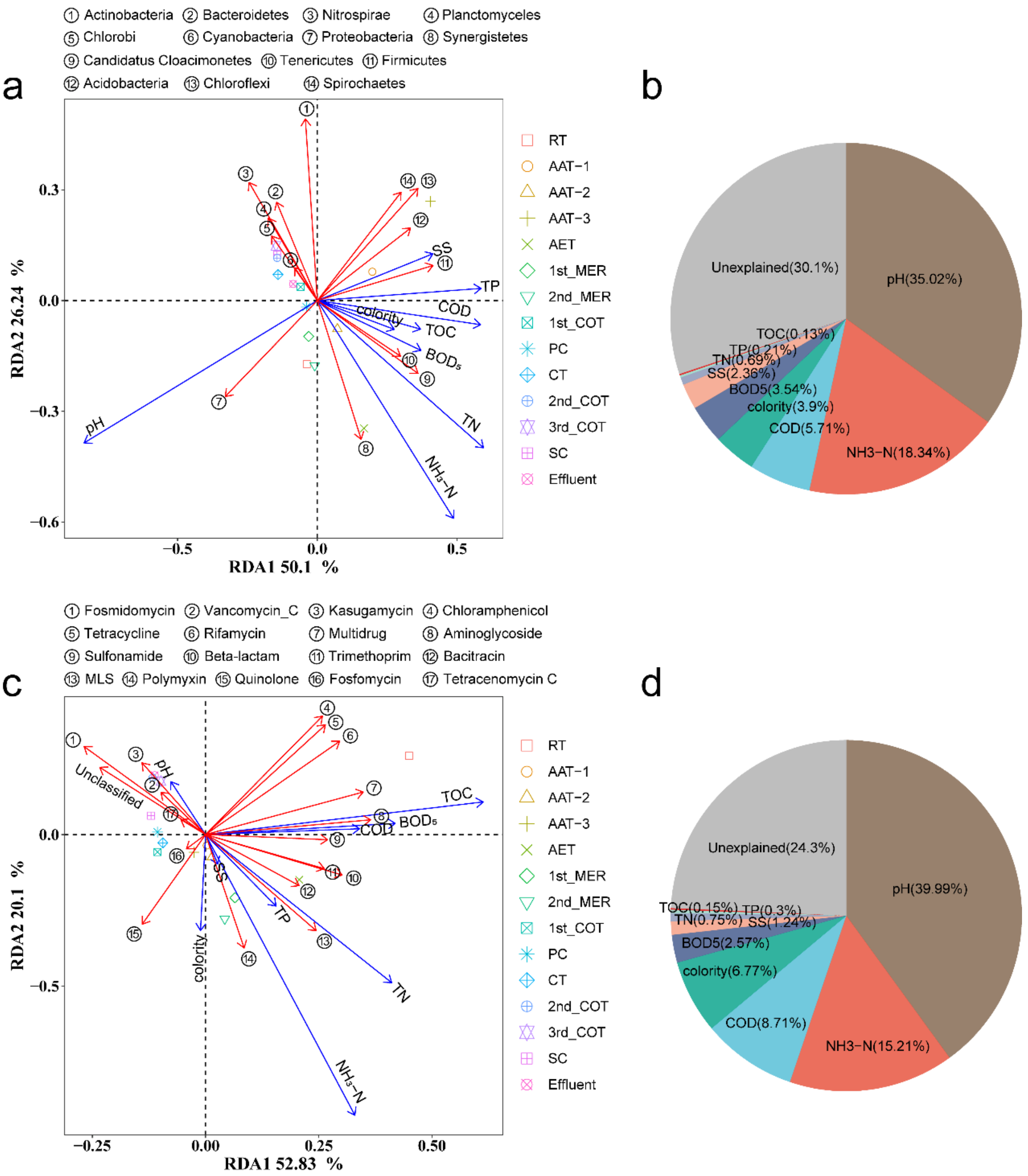

Figure S4. RDA analysis of the relationships between environment variables with (a) bacteria and (c) ARGs, respectively. Red arrows represent the phylum level of bacteria and blue arrows represent environment factors. Total explained variance ratio were calculated and visualized in (b) and (d), respectively.

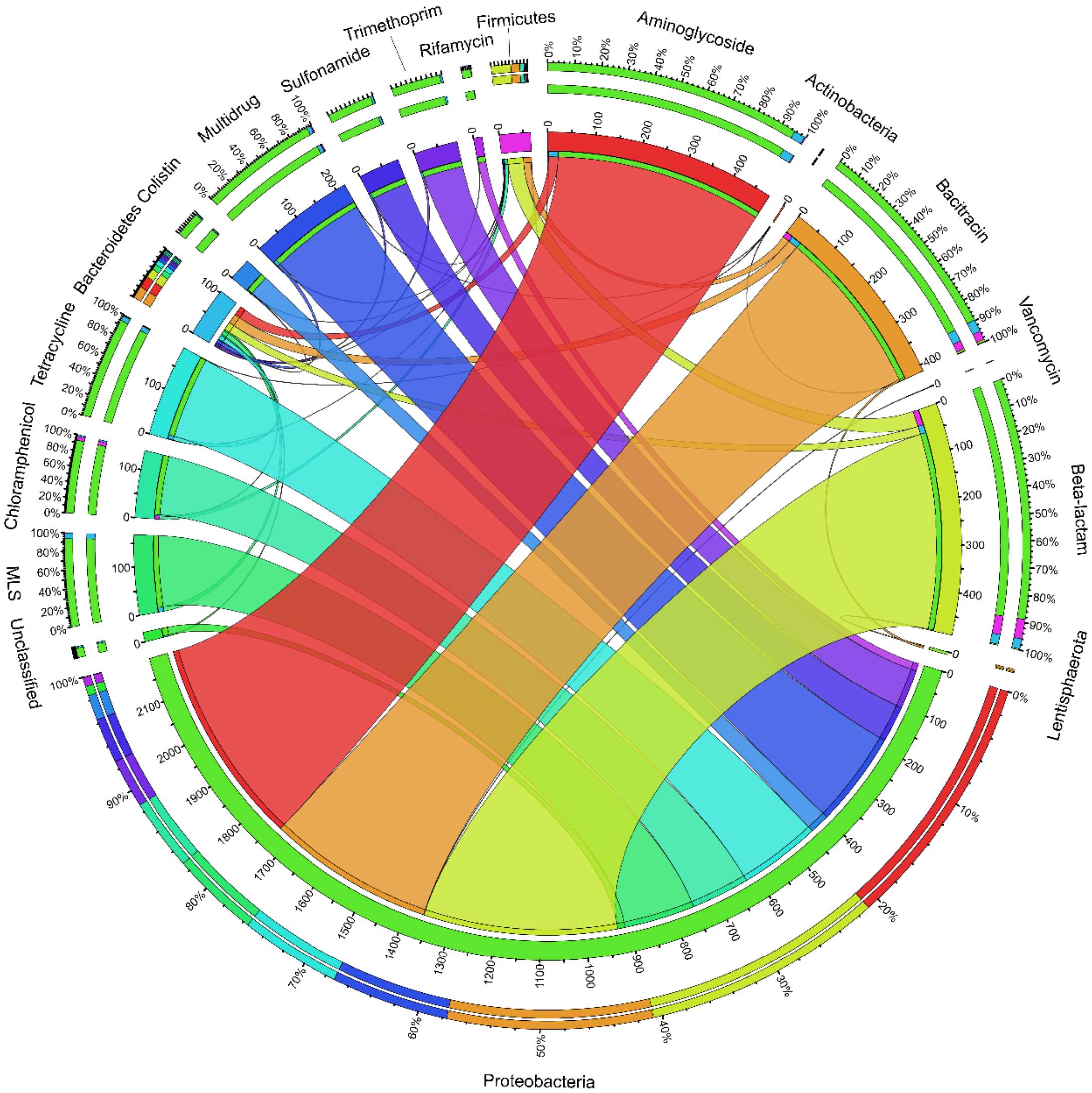

Figure S5. Hosts of ARGs at the phylum levels obtained from contigs statistical analysis.

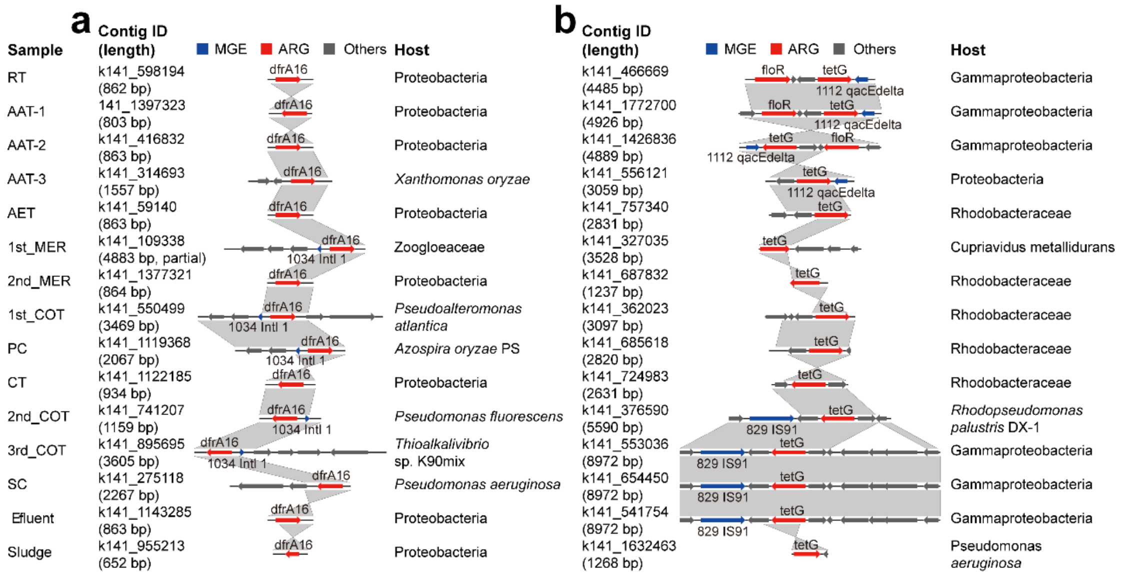

Figure S6. Co-shared (a) *dfrA16*-ARG-carrying and (b) *tetG*-ARG-carrying contigs and their putative hosts in different samples.

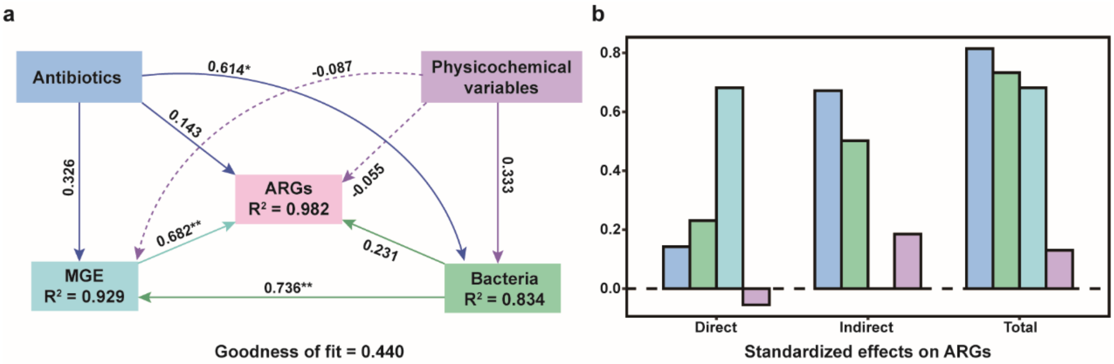

Figure S7. (a) The Partial least squares-path modeling analysis showing the effects of antibiotics, physicochemical variables, MGEs, and bacteria on ARG compositions. The solid and dashed lines indicate the positive and negative effects, respectively. The numbers adjacent to each arrow are partial correlation coefficients at the significance level of *** ≤ 0.001, ** ≤ 0.01, or * ≤ 0.05. The R^2 values represent the proportion of variance explained for each factor. (b) The standardized total effect of antibiotics, physicochemical variables, MGEs, and bacteria on the ARG compositions.

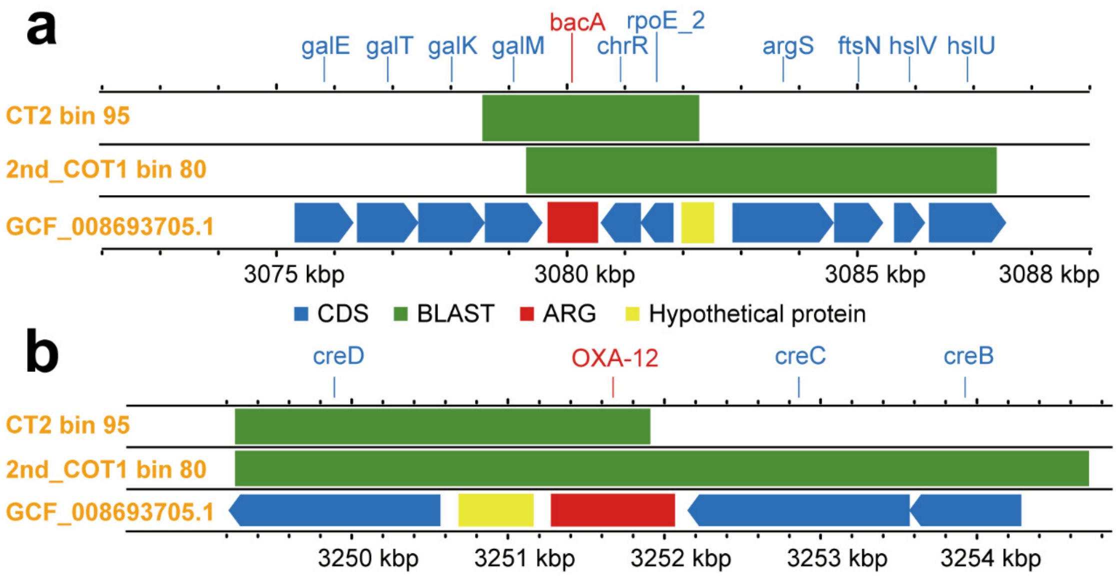

Figure S8. Comparisons of (a) *bacA* and (b) *OXA-12* genes in two assembled *A. veronii* MAGs and *A. veronii* reference genome.

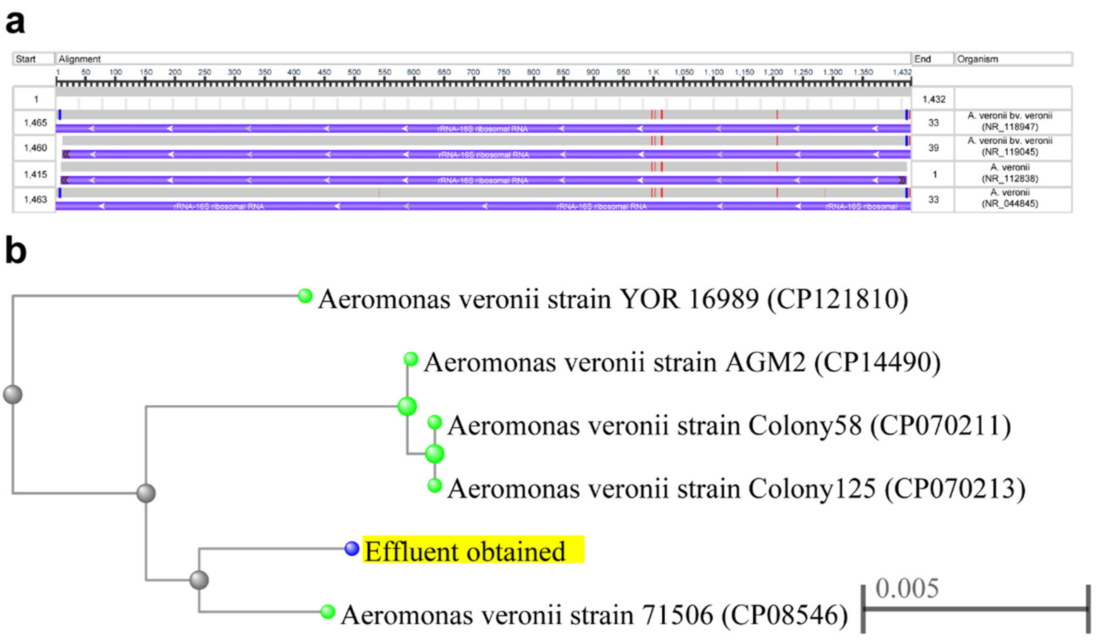

Figure S9. (a) NCBI Multiple Sequence Alignment of the 16S rRNA gene of the *A. veronii* obtained from the effluent and the most similar sequences. (b) Unrooted phylogenetic tree constructed by partial *ropB* gene of effluent obtained and some other isolated *A. veronii* strains.

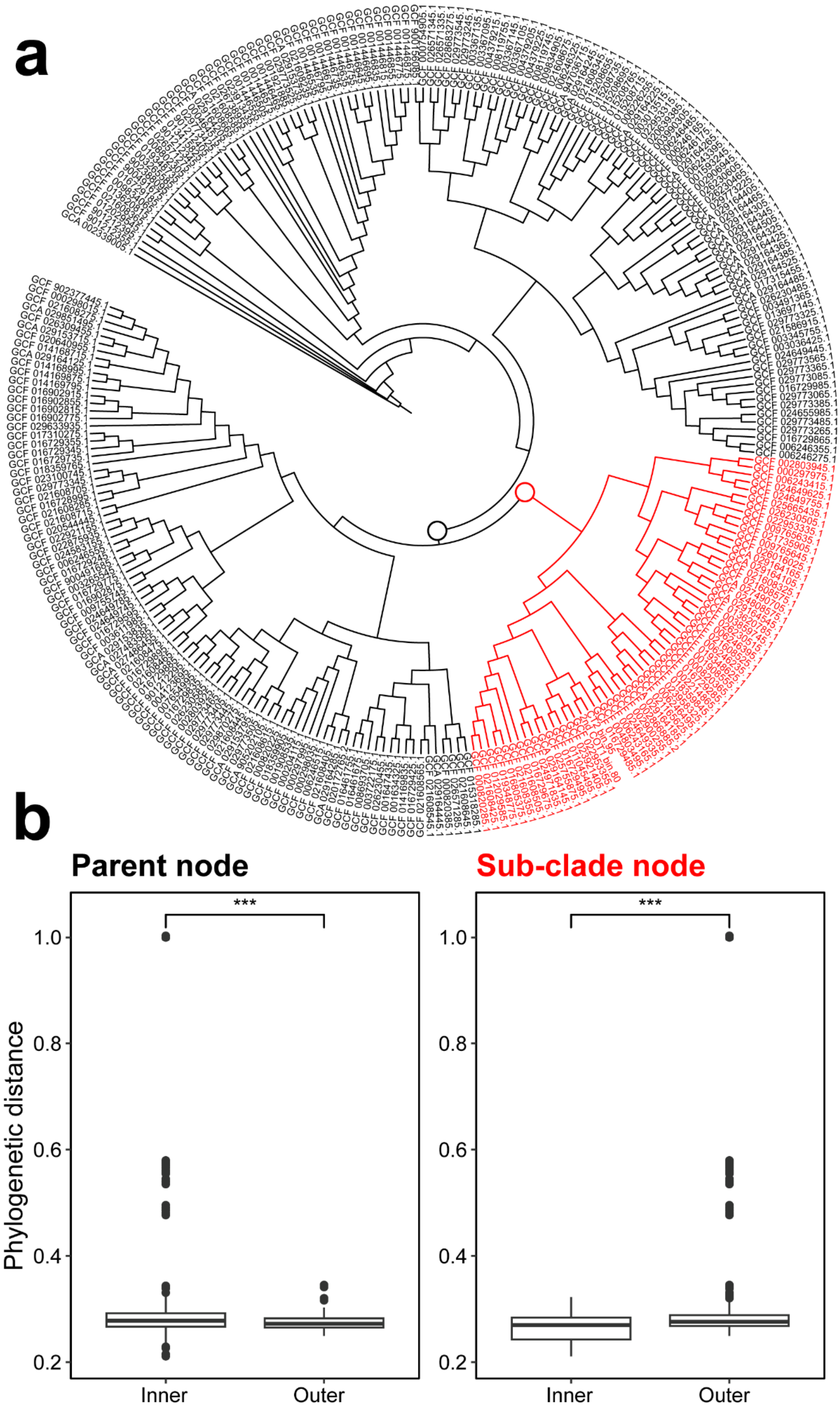

Figure S10. (a) Branch length ignored phylogenetic tree to show inner nodes clearly. Nodes used

in figure b is shown with circle. (b) Welch Two Sample t-test result of phylogenetic distance for genomes in sub-clade 1 and sub-clade 2 (right) and result of the parent node at the next level (left).

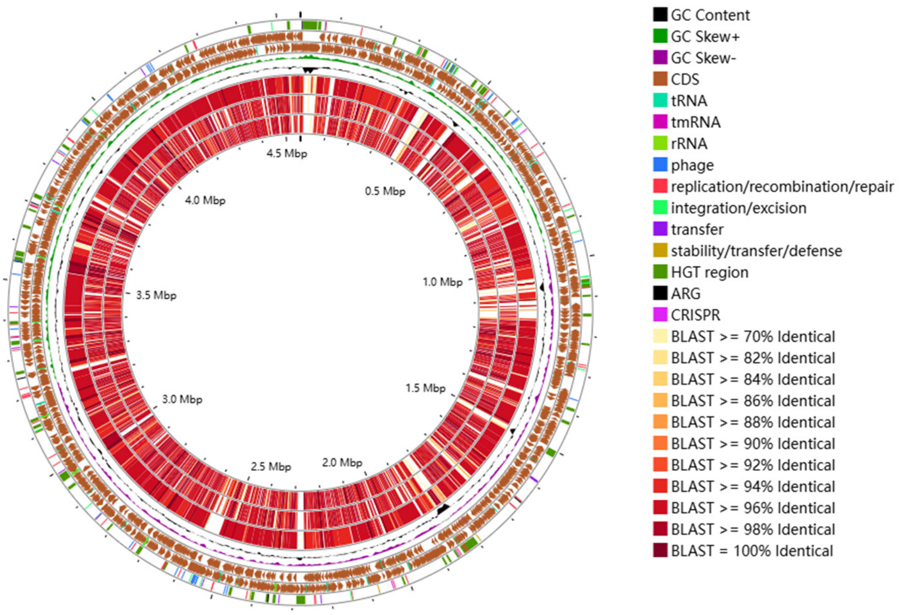

Figure S11. The circos map of A. veronii genomes' chromosome. Rings from inside to outside are: genome alignment results of (1) 2nd COT1 bin.80 vs reference genome (FDAARGOS 632), (2) CT2 bin.95 vs reference genome and (3) genome from diarrhea patient (GCF 016729485.1) vs reference genome; GC content of reference genome; GC skew of reference genome; genome annotation (+); genome annotation (-); positions related to ARG or HGT.

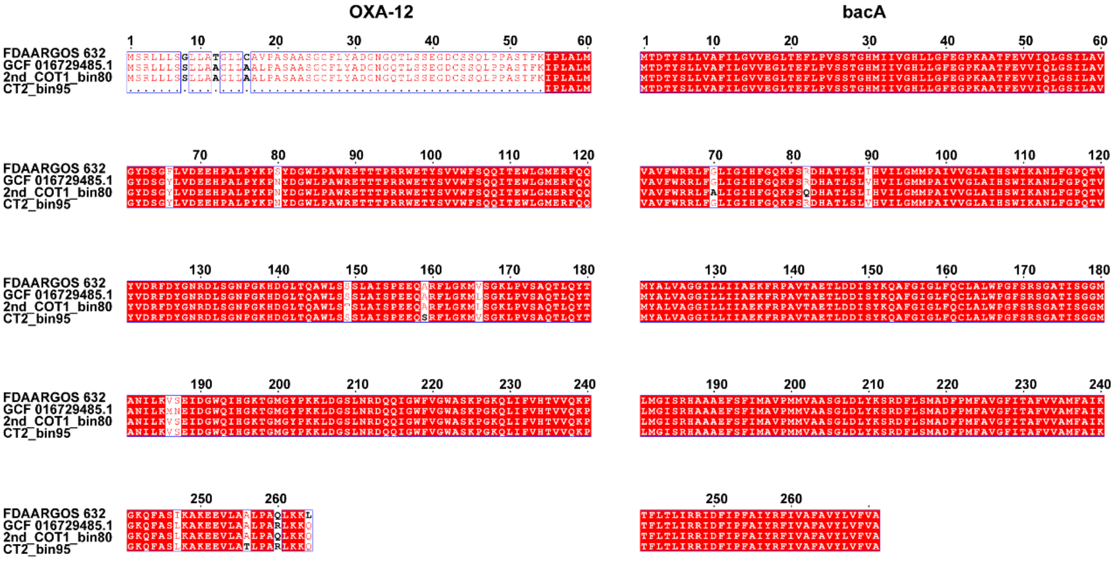

Figure S12. Multiple sequence alignment result for protein of *OXA-12* and *bacA*.

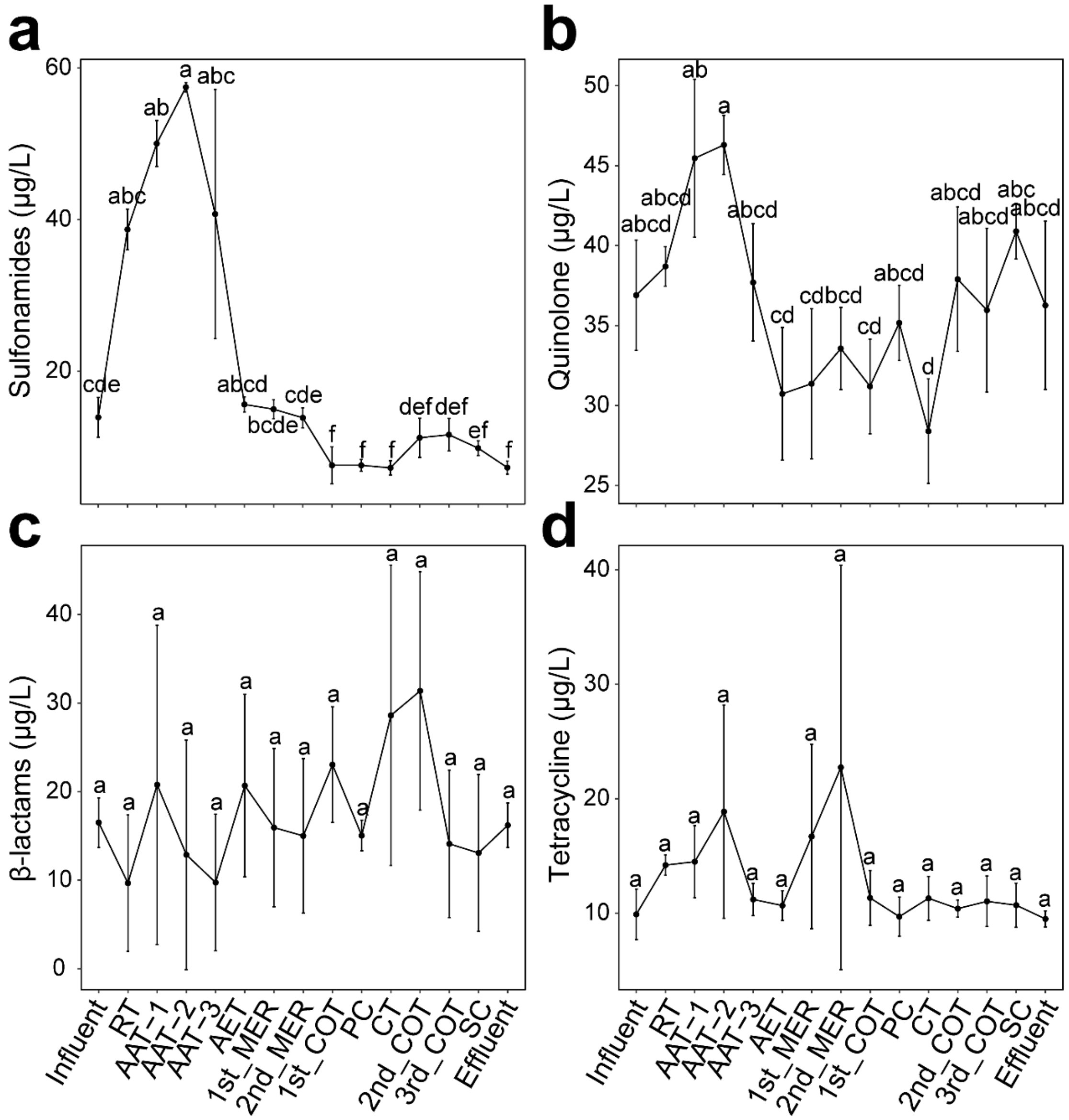

Figure S13. Concentration of the top 4 antibiotics in all wastewater samples. (a) Sulfonamides, (b) Quinolones, (c) *β*-lactams, and (d) Tetracyclines. Significance of differences is showed by letter. Error bars represent for Standard Deviation.

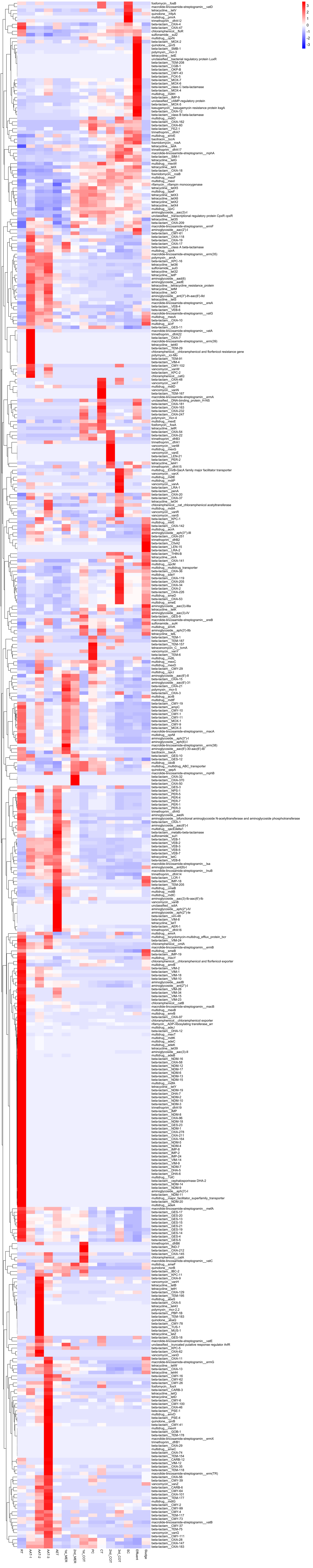

Additional file 1. Detailed ARG profiles in all samples.

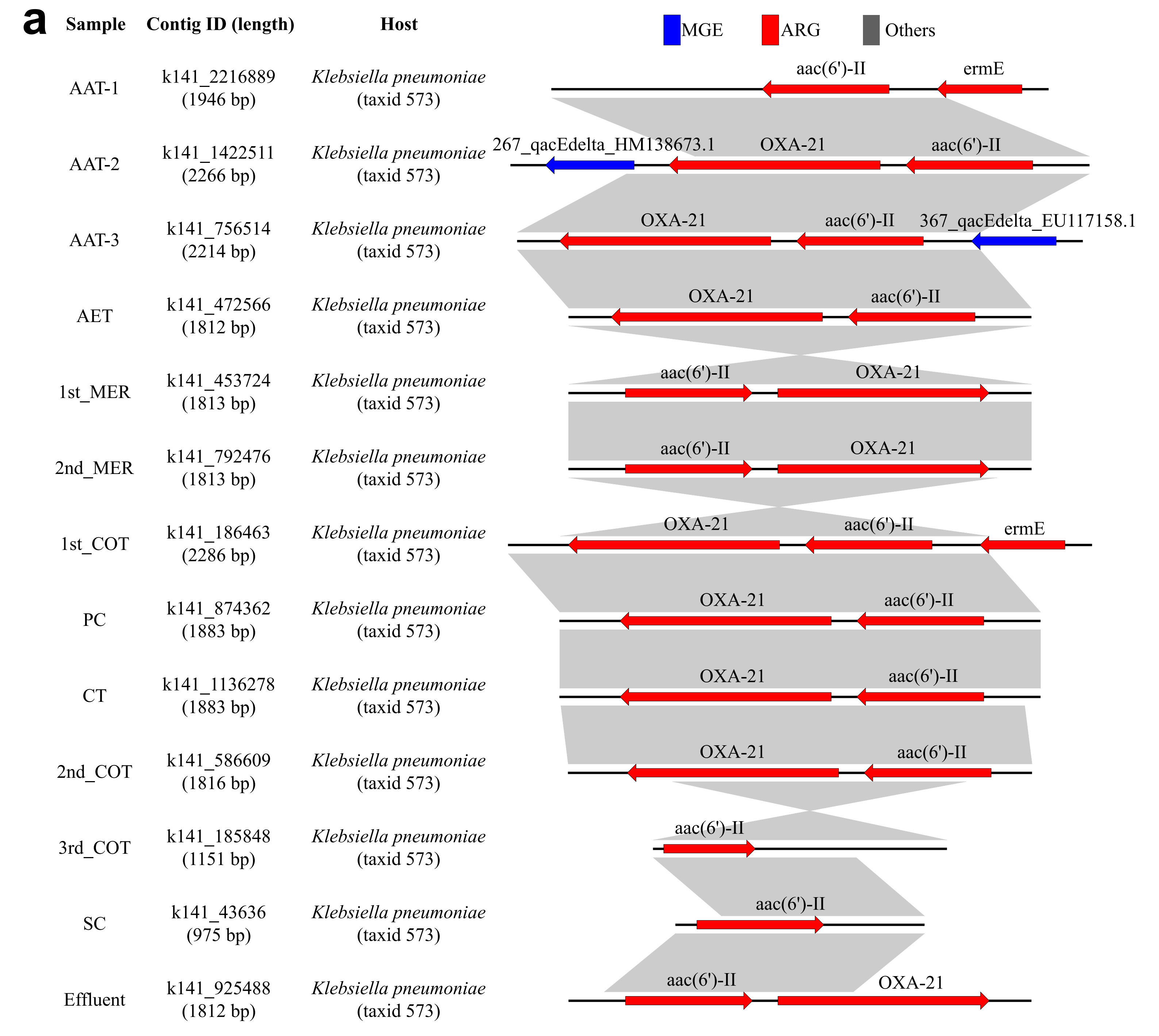
a
Sample
Contig ID (length)
Host
MGE
ARG
Others
AAT-1
k141_2216889 (1946 bp)
Klebsiella pneumoniae (taxid 573)
aac(6')-II
ermE
AAT-2
k141_1422511 (2266 bp)
Klebsiella pneumoniae (taxid 573)
267_qacEdelta_HM138673.1
OXA-21
aac(6')-II
AAT-3
k141_756514 (2214 bp)
Klebsiella pneumoniae (taxid 573)
OXA-21
aac(6')-II
367_qacEdelta_EU117158.1
AET
k141_472566 (1812 bp)
Klebsiella pneumoniae (taxid 573)
OXA-21
aac(6')-II
1st_MER
k141_453724 (1813 bp)
Klebsiella pneumoniae (taxid 573)
aac(6')-II
OXA-21
2nd_MER
k141_792476 (1813 bp)
Klebsiella pneumoniae (taxid 573)
aac(6')-II
OXA-21
1st_COT
k141_186463 (2286 bp)
Klebsiella pneumoniae (taxid 573)
OXA-21
aac(6')-II
ermE
PC
k141_874362 (1883 bp)
Klebsiella pneumoniae (taxid 573)
OXA-21
aac(6')-II
CT
k141_1136278 (1883 bp)
Klebsiella pneumoniae (taxid 573)
OXA-21
aac(6')-II
2nd_COT
k141_586609 (1816 bp)
Klebsiella pneumoniae (taxid 573)
OXA-21
aac(6')-II
3rd_COT
k141_185848 (1151 bp)
Klebsiella pneumoniae (taxid 573)
aac(6')-II
SC
k141_43636 (975 bp)
Klebsiella pneumoniae (taxid 573)
aac(6')-II
Effluent
k141_925488 (1812 bp)
Klebsiella pneumoniae (taxid 573)
aac(6')-II
OXA-21

b

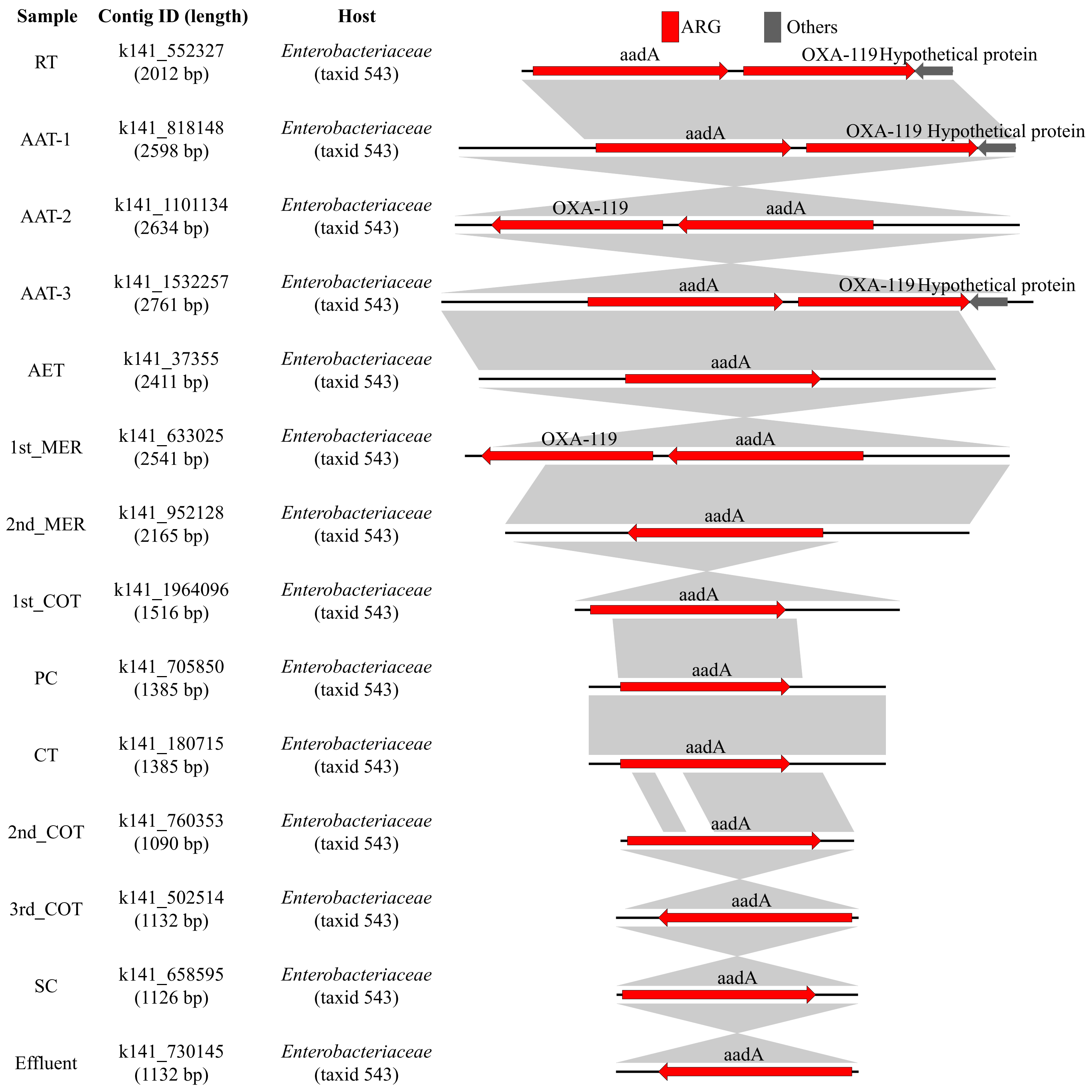
Sample
Contig ID (length)
Host
ARG
Others
RT
k141_552327 (2012 bp)
Enterobacteriaceae (taxid 543)
aadA
OXA-119
Hypothetical protein
AAT-1
k141_818148 (2598 bp)
Enterobacteriaceae (taxid 543)
aadA
OXA-119
Hypothetical protein
AAT-2
k141_1101134 (2634 bp)
Enterobacteriaceae (taxid 543)
OXA-119
aadA
AAT-3
k141_1532257 (2761 bp)
Enterobacteriaceae (taxid 543)
aadA
OXA-119
Hypothetical protein
AET
k141_37355 (2411 bp)
Enterobacteriaceae (taxid 543)
aadA
1st_MER
k141_633025 (2541 bp)
Enterobacteriaceae (taxid 543)
OXA-119
aadA
2nd_MER
k141_952128 (2165 bp)
Enterobacteriaceae (taxid 543)
aadA
1st_COT
k141_1964096 (1516 bp)
Enterobacteriaceae (taxid 543)
aadA
PC
k141_705850 (1385 bp)
Enterobacteriaceae (taxid 543)
aadA
CT
k141_180715 (1385 bp)
Enterobacteriaceae (taxid 543)
aadA
2nd_COT
k141_760353 (1090 bp)
Enterobacteriaceae (taxid 543)
aadA
3rd_COT
k141_502514 (1132 bp)
Enterobacteriaceae (taxid 543)
aadA
SC
k141_658595 (1126 bp)
Enterobacteriaceae (taxid 543)
aadA
Effluent
k141_730145 (1132 bp)
Enterobacteriaceae (taxid 543)
aadA

c

Sample	Contig ID (length)	Host
RT	k141_185903 (33194 bp)	*Thauera* sp. MZ1T (taxid 85643)
AAT-1	k141_2114621 (37882 bp)	*Thauera* sp. MZ1T (taxid 85643)
AAT-2	k141_886439 (36312 bp)	*Thauera* sp. MZ1T (taxid 85643)
AAT-3	k141_752109 (39351 bp)	*Thauera* sp. MZ1T (taxid 85643)
AET	k141_229968 (31888 bp)	*Thauera* sp. MZ1T (taxid 85643)
1st_MER	k141_1158421 (41228 bp)	*Thauera* sp. MZ1T (taxid 85643)
2nd_MER	k141_1574621 (40639 bp)	*Thauera* sp. MZ1T (taxid 85643)
1st_COT	k141_1472483 (65119 bp)	*Thauera* sp. MZ1T (taxid 85643)
PC	k141_1151413 (72875 bp)	*Thauera* sp. MZ1T (taxid 85643)
CT	k141_1142924 (50069 bp)	*Thauera* sp. MZ1T (taxid 85643)
2nd_COT	k141_1129266 (66696 bp)	*Thauera* sp. MZ1T (taxid 85643)
3rd_COT	k141_959437 (1491 bp)	*Thauera* sp. MZ1T (taxid 85643)
SC	k141_251617 (47375 bp)	*Thauera* sp. MZ1T (taxid 85643)
Effluent	k141_1117885 (33034 bp)	*Thauera* sp. MZ1T (taxid 85643)

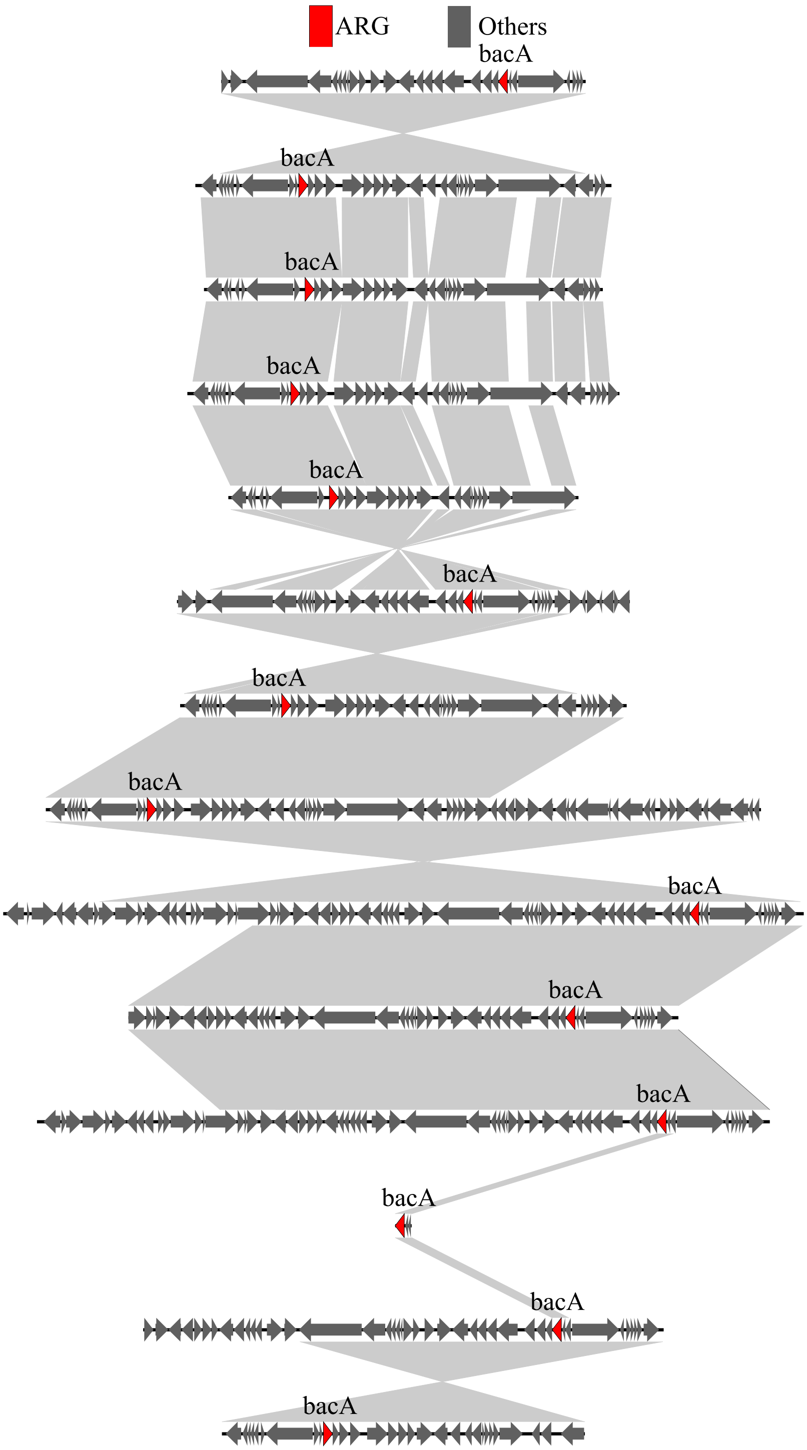

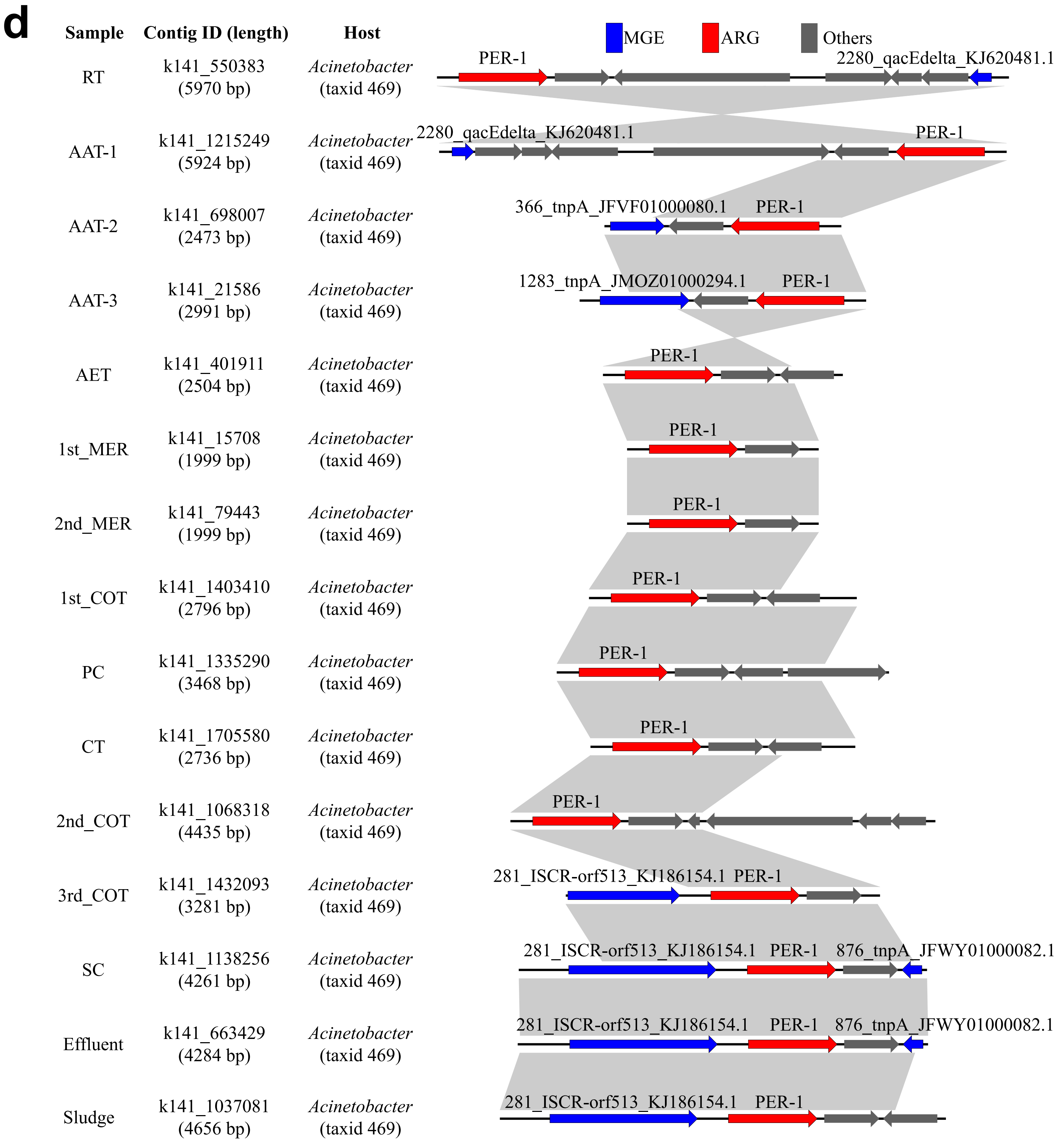

d
Sample
Contig ID (length)
Host
MGE
ARG
Others
RT
k141_550383 (5970 bp)
Acinetobacter (taxid 469)
PER-1
2280_qacEdelta_KJ620481.1
AAT-1
k141_1215249 (5924 bp)
Acinetobacter (taxid 469)
2280_qacEdelta_KJ620481.1
PER-1
AAT-2
k141_698007 (2473 bp)
Acinetobacter (taxid 469)
366_tnpA_JFVF01000080.1
PER-1
AAT-3
k141_21586 (2991 bp)
Acinetobacter (taxid 469)
1283_tnpA_JMOZ01000294.1
PER-1
AET
k141_401911 (2504 bp)
Acinetobacter (taxid 469)
PER-1
1st_MER
k141_15708 (1999 bp)
Acinetobacter (taxid 469)
PER-1
2nd_MER
k141_79443 (1999 bp)
Acinetobacter (taxid 469)
PER-1
1st_COT
k141_1403410 (2796 bp)
Acinetobacter (taxid 469)
PER-1
PC
k141_1335290 (3468 bp)
Acinetobacter (taxid 469)
PER-1
CT
k141_1705580 (2736 bp)
Acinetobacter (taxid 469)
PER-1
2nd_COT
k141_1068318 (4435 bp)
Acinetobacter (taxid 469)
PER-1
3rd_COT
k141_1432093 (3281 bp)
Acinetobacter (taxid 469)
281_ISCR-orf513_KJ186154.1
PER-1
SC
k141_1138256 (4261 bp)
Acinetobacter (taxid 469)
281_ISCR-orf513_KJ186154.1
PER-1
876_tnpA_JFWY01000082.1
Effluent
k141_663429 (4284 bp)
Acinetobacter (taxid 469)
281_ISCR-orf513_KJ186154.1
PER-1
876_tnpA_JFWY01000082.1
Sludge
k141_1037081 (4656 bp)
Acinetobacter (taxid 469)
281_ISCR-orf513_KJ186154.1
PER-1

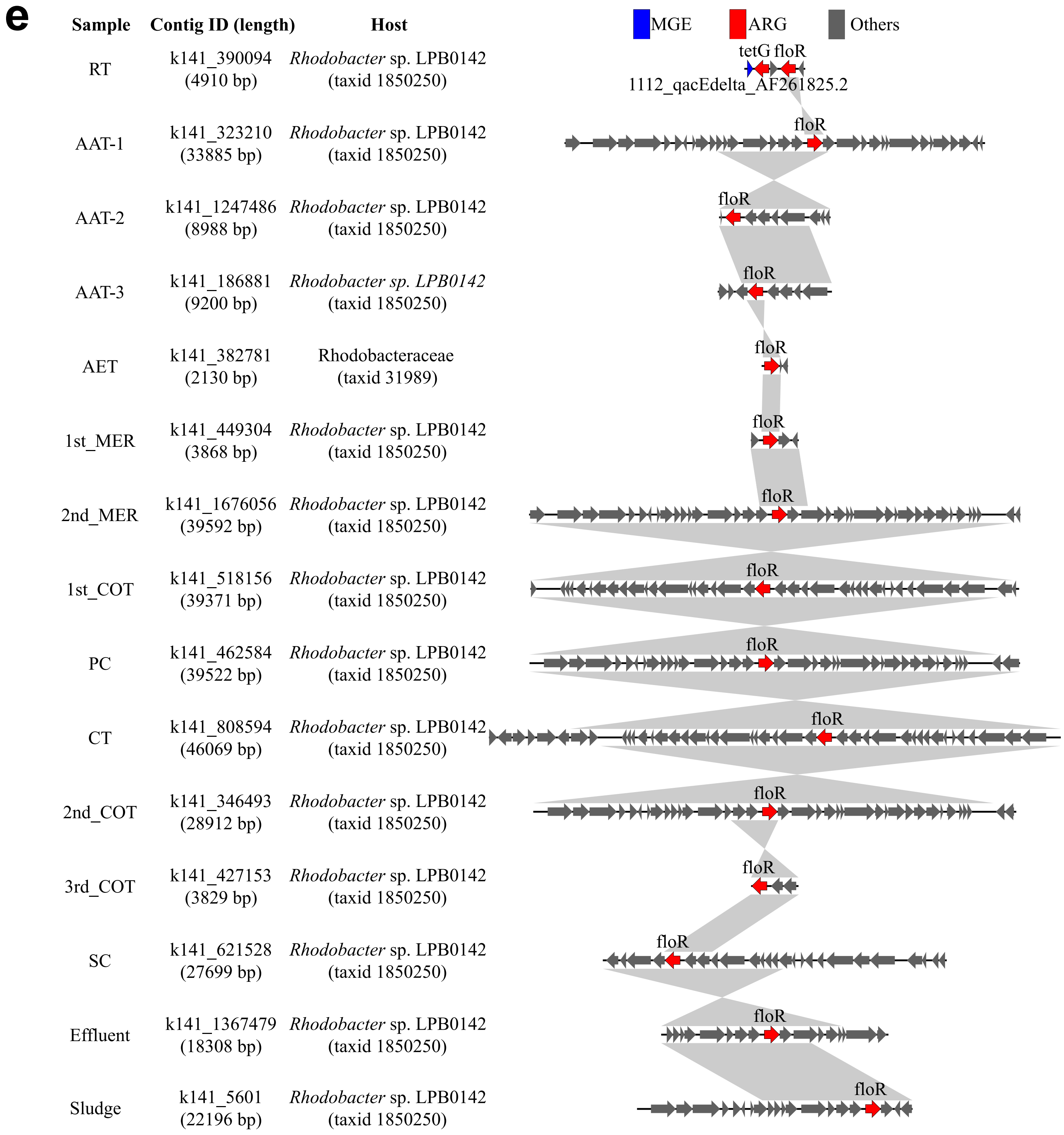
e
Sample
Contig ID (length)
Host
MGE
ARG
Others
RT
k141_390094 (4910 bp)
Rhodobacter sp. LPB0142 (taxid 1850250)
tetG
floR
1112_qacEdelta_AF261825.2
AAT-1
k141_323210 (33885 bp)
Rhodobacter sp. LPB0142 (taxid 1850250)
floR
AAT-2
k141_1247486 (8988 bp)
Rhodobacter sp. LPB0142 (taxid 1850250)
floR
AAT-3
k141_186881 (9200 bp)
Rhodobacter sp. LPB0142 (taxid 1850250)
floR
AET
k141_382781 (2130 bp)
Rhodobacteraceae (taxid 31989)
floR
1st_MER
k141_449304 (3868 bp)
Rhodobacter sp. LPB0142 (taxid 1850250)
floR
2nd_MER
k141_1676056 (39592 bp)
Rhodobacter sp. LPB0142 (taxid 1850250)
floR
1st_COT
k141_518156 (39371 bp)
Rhodobacter sp. LPB0142 (taxid 1850250)
floR
PC
k141_462584 (39522 bp)
Rhodobacter sp. LPB0142 (taxid 1850250)
floR
CT
k141_808594 (46069 bp)
Rhodobacter sp. LPB0142 (taxid 1850250)
floR
2nd_COT
k141_346493 (28912 bp)
Rhodobacter sp. LPB0142 (taxid 1850250)
floR
3rd_COT
k141_427153 (3829 bp)
Rhodobacter sp. LPB0142 (taxid 1850250)
floR
SC
k141_621528 (27699 bp)
Rhodobacter sp. LPB0142 (taxid 1850250)
floR
Effluent
k141_1367479 (18308 bp)
Rhodobacter sp. LPB0142 (taxid 1850250)
floR
Sludge
k141_5601 (22196 bp)
Rhodobacter sp. LPB0142 (taxid 1850250)
floR

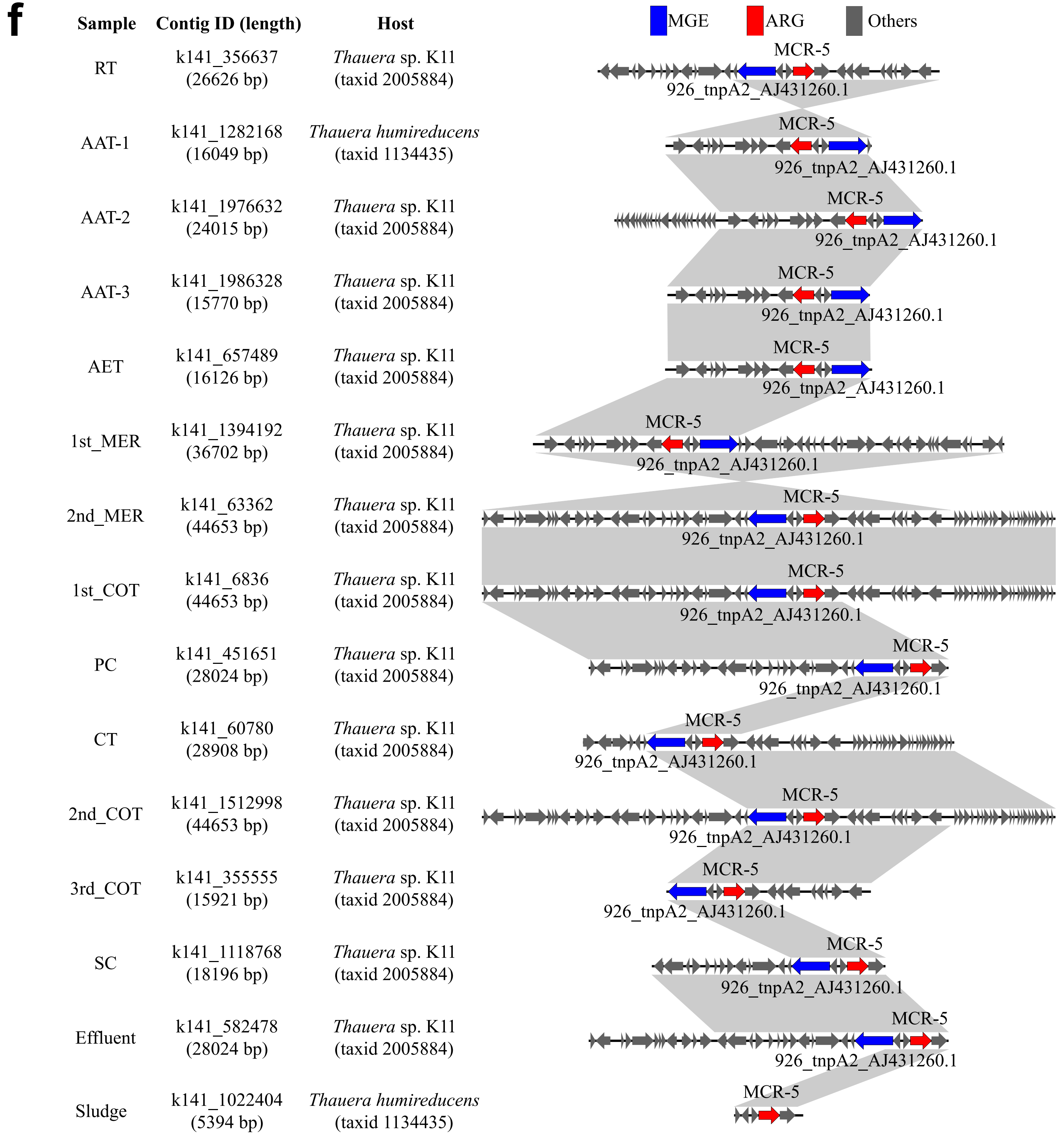
f
Sample
Contig ID (length)
Host
MGE
ARG
Others
RT
k141_356637 (26626 bp)
Thauera sp. K11 (taxid 2005884)
AAT-1
k141_1282168 (16049 bp)
Thauera humireducens (taxid 1134435)
AAT-2
k141_1976632 (24015 bp)
Thauera sp. K11 (taxid 2005884)
AAT-3
k141_1986328 (15770 bp)
Thauera sp. K11 (taxid 2005884)
AET
k141_657489 (16126 bp)
Thauera sp. K11 (taxid 2005884)
1st_MER
k141_1394192 (36702 bp)
Thauera sp. K11 (taxid 2005884)
2nd_MER
k141_63362 (44653 bp)
Thauera sp. K11 (taxid 2005884)
1st_COT
k141_6836 (44653 bp)
Thauera sp. K11 (taxid 2005884)
PC
k141_451651 (28024 bp)
Thauera sp. K11 (taxid 2005884)
CT
k141_60780 (28908 bp)
Thauera sp. K11 (taxid 2005884)
2nd_COT
k141_1512998 (44653 bp)
Thauera sp. K11 (taxid 2005884)
3rd_COT
k141_355555 (15921 bp)
Thauera sp. K11 (taxid 2005884)
SC
k141_1118768 (18196 bp)
Thauera sp. K11 (taxid 2005884)
Effluent
k141_582478 (28024 bp)
Thauera sp. K11 (taxid 2005884)
Sludge
k141_1022404 (5394 bp)
Thauera humireducens (taxid 1134435)
MCR-5
926_tnpA2_AJ431260.1

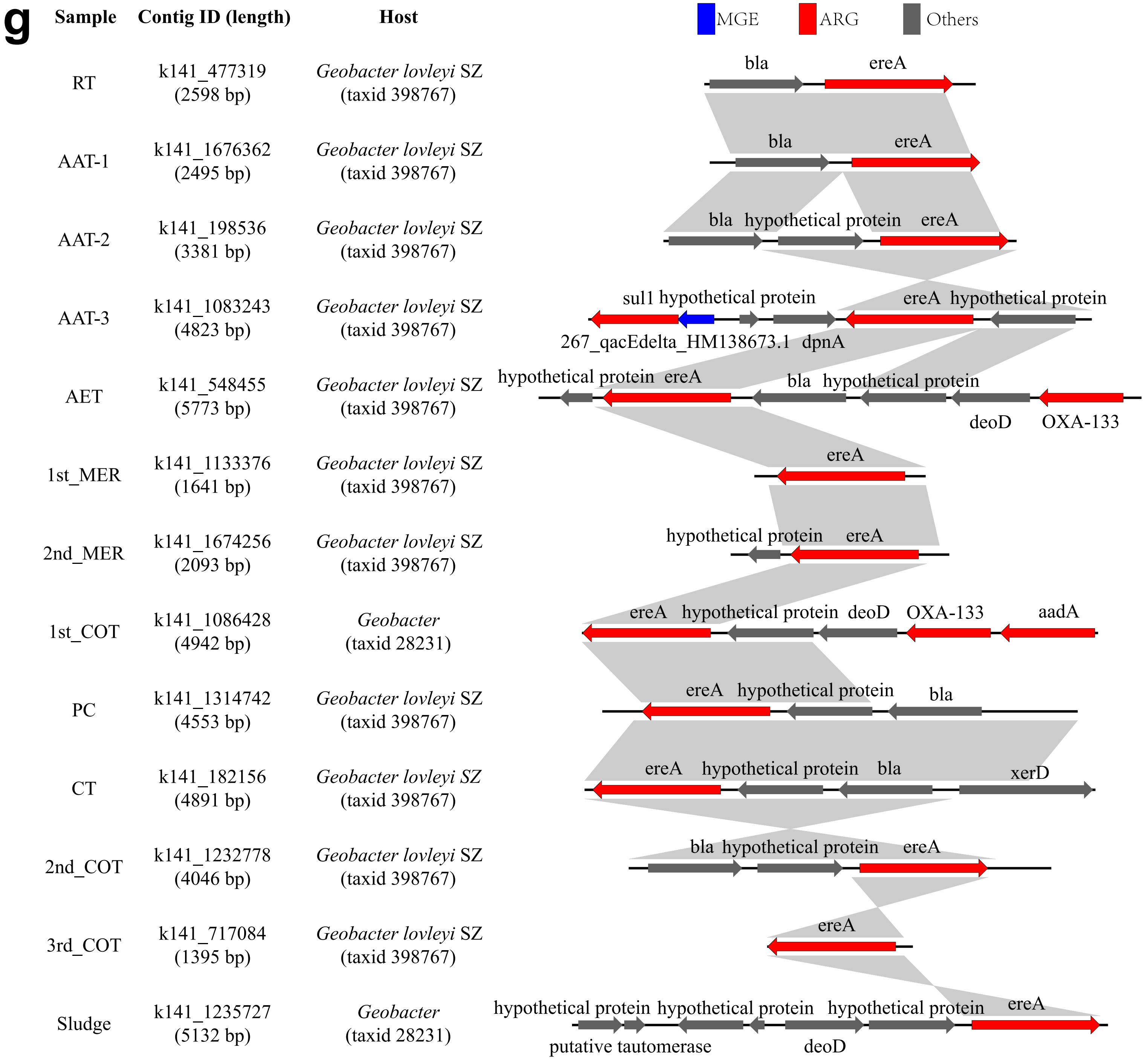

Additional file 2. (a-g) Some ARGs with identical host.
